# A DNA nanoscope via auto-cycling proximity recording

**DOI:** 10.1038/s41467-017-00542-3

**Published:** 2017-09-25

**Authors:** Thomas E. Schaus, Sungwook Woo, Feng Xuan, Xi Chen, Peng Yin

**Affiliations:** 1 Wyss Institute for Biologically Inspired Engineering, Harvard University, 3 Blackfan Circle, 5th Floor, Boston, MA 02115 USA; 2 Department of Systems Biology, Harvard Medical School, Boston, MA 02115 USA

## Abstract

Analysis of the spatial arrangement of molecular features enables the engineering of synthetic nanostructures and the understanding of natural ones. The ability to acquire a comprehensive set of pairwise proximities between components would satisfy an increasing interest in investigating individual macromolecules and their interactions, but current biochemical techniques detect only a single proximity partner per probe. Here, we present a biochemical DNA nanoscopy method that records nanostructure features in situ and in detail for later readout. Based on a conceptually novel auto-cycling proximity recording (APR) mechanism, it continuously and repeatedly produces proximity records of any nearby pairs of DNA-barcoded probes, at physiological temperature, without altering the probes themselves. We demonstrate the production of dozens of records per probe, decode the spatial arrangements of 7 unique probes in a homogeneous sample, and repeatedly sample the same probes in different states.

## Abstract (web summary)

The spatial organisation of nanostructures is fundamental to their function. Here, the authors develop a non-destructive, proximity-based method to record extensive spatial organization information in DNA molecules for later readout.

## Introduction

Spatial organization of molecular components is fundamental to the function of synthetic^1–5^ and biological^6–8^ nanostructures. This organization can be described as a set of pairwise proximities between the components. Single-molecule methods to study proximity and organization must examine individual nanostructures with molecular-scale precision, and are foundational to advancing nanoscience^9–12^.

Though limited to multiplexing a modest number of simultaneous species, direct visualization by electron, atomic force, and optical microscopy have identified individual macromolecular associations in synthetic^4, 5, 13^ and biological^14–16^ systems, and achieved molecular resolution in controlled, static environments^17^. Resonance energy transfer (FRET) techniques further allow for dynamic measurement of pairwise proximity in solution^18, 19^. Complementary to microscopy are the biochemical techniques that have enabled ensemble measurements of protein interaction networks with molecular precision, including yeast two-hybrid^20–22^ and related^23^ assays, affinity purification/mass spectroscopy^24^, and co-immunoprecipitation^25^. They are relatively easily parallelized, executed, and even automated.

Barcoding with DNA has recently given biochemical techniques the potential for single-molecule precision. Proximity ligation assay (PLA)^26^ or proximity extension assay (PEA)^27^, which record the colocalization of two probes by ligating or extending probe-bound DNA sequences, now routinely multiplex ~100 signals with orthogonal sequences. The information content of DNA itself is much larger, however. There are 4^*N*^ combinations of *N* nucleotides, enabling 1 million-plex with only 10-nucleotide strings. Unfortunately, because ligated or extended probes are depleted in detecting a single pairwise association, no more than a single association per molecular target can be recorded. This makes it difficult to reconstruct complexes on a single-molecule basis.

Here, we present a new biochemical interrogation technique that records nanostructure features in situ and in detail for later readout. Termed auto-cycling proximity recording (APR), it repeatedly produces proximity records of any nearby pairs of DNA-barcoded probes, without altering the probes themselves. We describe and characterize the mechanism in detail, and then demonstrate a biochemical DNA nanoscope that decodes the complex spatial arrangements of seven unique APR probes in a homogeneous sample. We further show that different states of the same probe set can be repeatedly interrogated.

Although we used PCR and gel-based assays on homogeneous samples to demonstrate the nanoscope, APR can in principle be applied with uniquely barcoded probes and read with massively parallel sequencing. We expect that further development of small-sample sequencing pipelines^28^ and computational analysis will enable APR to achieve massively parallel, single-molecule precision.

## Results

### The APR concept

**Fig. 1:**
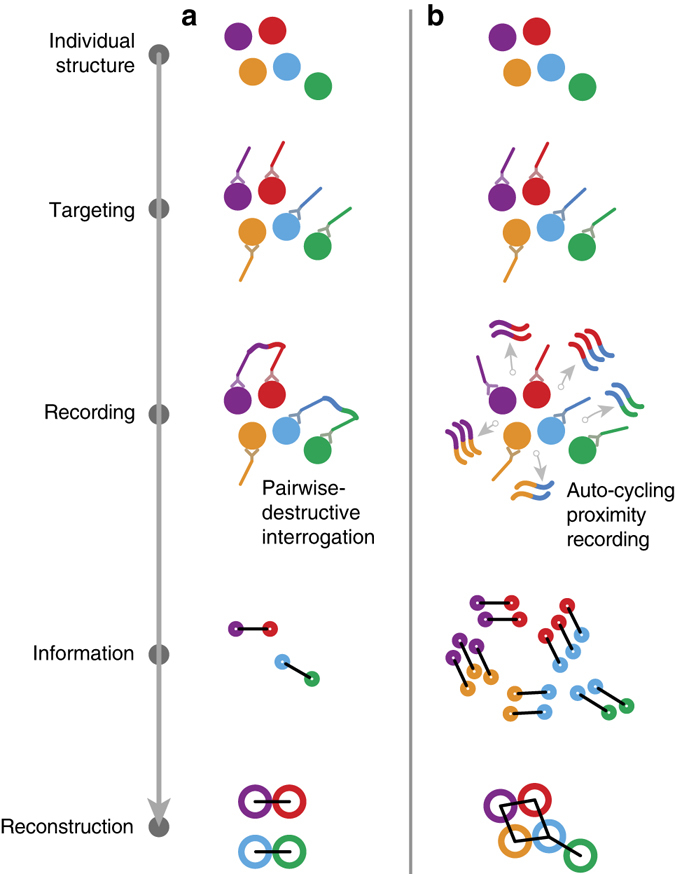
Comparison between existing pairwise-destructive proximity measurements and auto-cycling proximity recording (APR). **a** In pairwise-destructive methods (e.g., proximity ligation), a particular target-bound probe generates a proximity record with only one neighbor that it becomes permanently attached to. This in turn yields only limited proximity information for a particular multitarget structure, necessarily resulting in incomplete reconstruction of its target arrangement. **b** With APR, proximity records are generated autonomously and continuously by transient pairing of any nearby probes, without destroying or depleting them. Not only are multiple copies of a record generated for a particular pair of probes, improving signal, but each probe can also produce proximity records with every neighbor, potentially allowing for complete reconstruction

Figure 1a describes the inherent limitations of current, pairwise-destructive methods in the amount of information recorded from an individual structure. Because only a single proximity is recorded from each probe, proximity information remains isolated in unconnected pairs and reconstruction is incomplete. In contrast, APR (Fig. 1b) generates proximity data autonomously and repeatedly, at tunable distances, by nondestructively copying pairs of DNA-barcoded probes. The result is a complete set of proximity information and complete reconstruction. The APR mechanism also provides for high signal levels, as well as allows resampling of the same molecular targets in different proximity arrangements.

### The APR mechanism

**Fig. 2:**
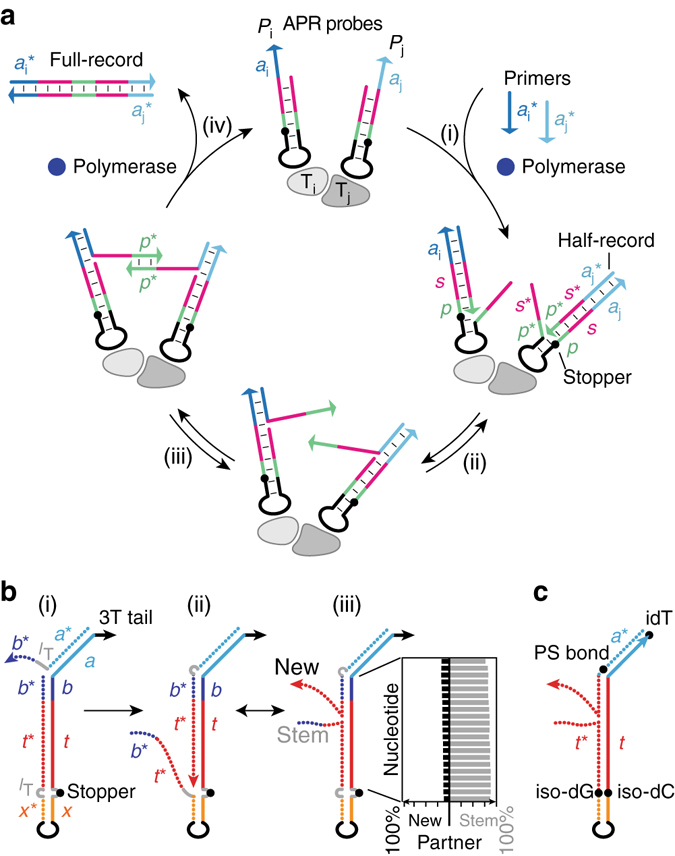
Auto-cycling proximity recording (APR) mechanisms. **a** The APR cycle creates molecular records of proximity from pairs of hairpin probes. Probe-specific primers are (step i in **a**) extended to Half-records and (ii) reversibly displaced, (iii) bind palindromic domains of nearby Half-records, and (iv) are extended on each other to create and release Full-records, regenerating the probes. See text for detailed description. **b** Copy-and-release hairpin (CRH) detail, depicting primer binding domain *b* explicitly but labeling the entire copied template simply as *t*. Shown is the mechanism of (state *i* in **b**) initial primer binding, (ii) extension, and (iii) random walk of the strand displacement branch. For this combination of primer bulge *l*_T_ and Spacer9 stopper shown, the hairpin template strand *t*−*b* is computationally predicted to pair predominantly with the hairpin stem complement (iii, plot). See also Supplementary Fig. 1. **c** A more rapidly cycling hairpin does not utilize bulge *l*_T_ or domain *b*, but instead uses a phosphorothioate (PS) bond before the final primer (*a*) nucleotide, in conjunction with synthetic nucleotide pair iso-dC/iso-dG as a stopper. See Supplementary Fig. 2 for sequences and Supplementary Note 1 for other considerations

The APR reaction proceeds in an autonomous, cyclic fashion, at physiologic conditions, to convert soluble primers into molecular records of probe pair proximity (Fig. 2a). As configured for ensemble applications, DNA hairpin probes *P*_i_ and *P*_j_ are first attached to target molecules *T*_i_ and *T*_j_ via antibodies, aptamers, direct nucleic acid hybridization, or other means, and encode the identity of the target type within unique primer binding sequences (labeled as sequence domains *a*_i_ and *a*_j_). In step (i) of Fig. 2a, the soluble primers (sequences $a_{\rm{i}}^{\rm{*}}$ and $a_{\rm{j}}^{\rm{*}}$, where the asterisk denotes a complement) stably bind their respective hairpin probe type and are extended by a polymerase through the spacer (*s*) and palindromic (*p*) domains up to the stopper site, thereby producing Half-records ($a_{\rm{i}}^{\rm{*}} - {s^{\rm{*}}} - {p^{\rm{*}}}$ and $a_{\rm{j}}^{\rm{*}} - {s^{\rm{*}}} - {p^{\rm{*}}}$). In step (ii), Half-records are partially displaced by spontaneously re-forming hairpin stems (${s^*}-{p^*}/p-s$) and (step iii) bind any nearby Half-records at their 3′ palindromic (${p^*}$) segments. These sequences are again extended in step (iv), this time over the partner spacer ${s^*}$ and primer ${a^*}$ segments, causing the release of Full-records that carry both probe identities as $a_{\rm{i}}^{\rm{*}}$ and $a_{\rm{j}}^{\rm{*}}$. Probes, thus regenerated to their initial state, may undergo additional cycles in the same or other pairings. Upon termination of this cyclic recording reaction, a specific Full-record species can be tested for by PCR amplification (with respective primer pair $a_{\rm{i}}^{\rm{*}}$, $a_{\rm{j}}^{\rm{*}}$) and gel electrophoresis. In this manner, for example, an isolated sample with PCR primers for *a*_i_ and *a*_j_ would yield a positive result in the presence of *T*_i_−*T*_j_ proximity.

This cycle solves two main challenges in generating multiple records between any nearby pairs. First, a template sequence (in Fig. 2a, ${s^*}-{p^*}$) must be copied from the probe and then spontaneously made single stranded and available for further reaction. Autonomous cycling requires that this proceeds without external thermal or chemical influence. Second, in the context of target proximity, each pair of copied sequences must be combined into a single molecular record and then released.

The first challenge is solved by a DNA-based copy-and-release hairpin (CRH) that enables the isothermal copying and release of an arbitrary sequence onto each primer strand (Fig. 2b). While simply extending a primer on a single-stranded template, as in PCR, would leave the product stably hybridized, here the template is bound to a complementary sequence that aids in its removal. The detailed mechanism for most experiments is as follows. In step (i), DNA primer strand ${a^*}-{l_{\rm T}}-{b^*}$ (i.e., of domains ${a^*}$ and ${b^*}$ with intervening single T nucleotide linker *l*_T_) binds the 3′ end of the hairpin at primer binding domain *a*. In step (ii), the primer and hairpin ${b^*}$ domains compete for short probe domain *b*. Primer domain ${b^*}$ occasionally binds, despite the resulting single T nucleotide *l*_T_ bulge, and is extended by a displacing polymerase (here, Bst, New England Biolabs, or Bsm, Thermo Fisher) through stem template domain *t*. When primer extension reaches a stopper modification near the loop (here, covalent triethylene glycol linker Spacer 9, Integrated DNA Technologies (IDT), opposite a single T *l*_T_), the polymerase dissociates. In step (iii), the new extension and the displaced hairpin stem, being identical in sequence (${b^*}-{t^*}$), rapidly compete for stem template domains *t*−*b* via the strand displacement mechanism^29^ with millisecond transit times^30, 31^. Computer models (NUPACK^32^) predict that the stem template *t*−*b* is more likely to be bound to its stem-based complement than to the new primer extension (iii, plot, as well as Supplementary Fig. 1), ensuring the Half-record is largely single-stranded and therefore available for further reaction. In contrast, the 16 nt length of sequence ${a^*}$ ensures that the Half-record remains stably bound until displaced by a polymerase.

The second APR design challenge, linking these Half-records to release a Full-Record in the context of proximity, is solved by copying a short, 6-nucleotide palindromic sequence *p* (ACGCGT or similar in Fig. 2b, or stronger GGCGCC in Fig. 2c) to the 3′ end of the Half-record. Here, a palindrome is defined such that $p \equiv {p^*}$, enabling any Half-record to bind to any other Half-record by their 3′ ends. Upon hybridization of two *p* sequences, extension by the polymerase copies the information of each Half-record onto the other, ultimately displacing and releasing the nascent Full-record from both probes. Note that nonspecific interaction between unbound Half-records is prevented by two features: the *p* domain is not part of the soluble primer but only added upon Half-record formation on a probe, and these Half-records are only released once they pair. See Supplementary Fig. 2 for further probe detail and sequences.

**Fig. 3:**
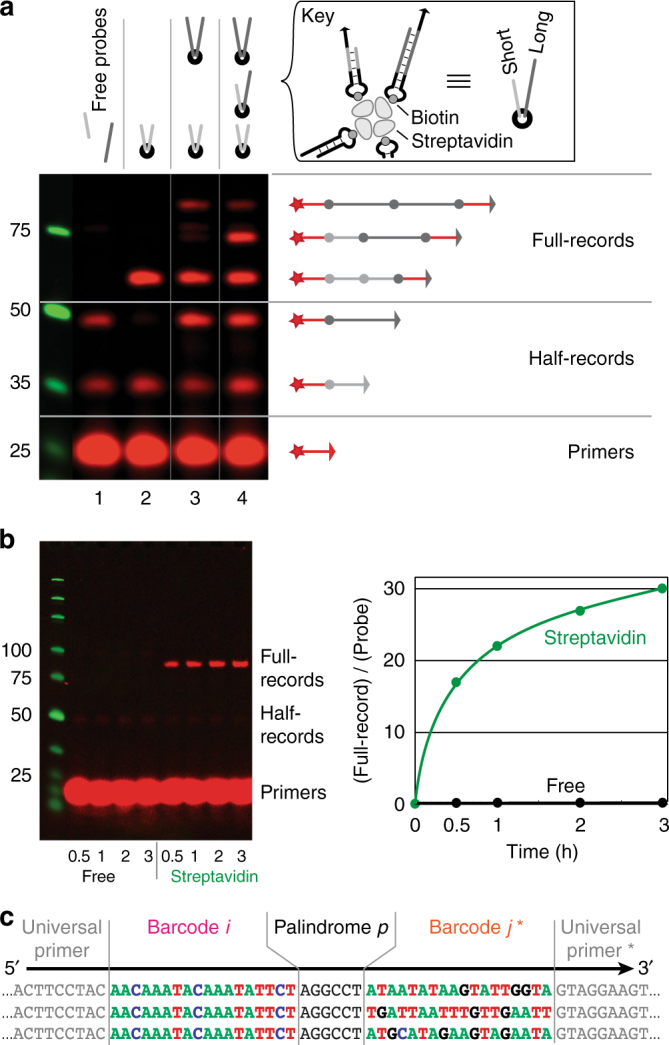
Proof-of-principle experiments. **a** Generation of Full-records requires the colocalization of probes, here by biotinylated hairpin loops bound to streptavidin, whereas isolated probes can always generate Half-records. Cropped denaturing PAGE gel depicting 10 μl reactions (40 min at 37 °C) with biotin–streptavidin association and 4:1 overall probe/streptavidin stoichiometry (*inset*), 8 and 22 nt barcodes (19 and 33 nt stem lengths copied), 10:1 initial primer/probe, and 40 nM total probe concentration. A single primer sequence was used and no secondary amplification was performed. **b** Auto-cycling is demonstrated by quantification of Cy5-labeled probes on cropped denaturing PAGE gels. Rapidly cycling probes with Iso-dC/dG stoppers and phosphorothioate primers were used, with probes at 0.1 nM and primers at 1000-fold excess to probes in each time series. Quantification of Full-records yields the plot. Half-records are difficult to detect because of low probe concentration. See probe details and sequences for **a**, **b** in Supplementary Fig. 2 and full gels in Supplementary Fig. 3. **c** An example of a single probe (with Barcode *i*) making multiple partnerships (with Barcodes ${j^*}$), read with Illumina MiSeq next-generation sequencing. Here, probes encoded a universal primer sequence and unique barcodes (in place of spacer *s* of Fig. 2a), and were held in tetramers by streptavidin. Primer sequences cropped for clarity. See Supplementary Fig. 4 for the unique-barcode APR cycle, probe details, and sequencing methods

Details of the CRH probe are important to reliability and cycling speed. Among the most important is the mechanism that biases the system toward a closed hairpin and single-stranded Half-record (${b^*}-{t^*}$), in which the cycle can progress. In Fig. 2b, depicting the probe design used in all figures except Fig. 3b, the stopper represents an internal loop^33^ in the hairpin duplex. Though the loop terminates polymerase extension, the one nucleotide (T) tether between hairpin complement ${t^*}$ and lower stem ${x^*}$ slows initiation of the strand displacement process that releases the Half-record. Left unbalanced by a similar effect at the primer end, this would result in a predominantly open hairpin and bound Half-record ${b^*}-{t^*}$. The *T* nucleotide bulge^33^ within primer ${a^*}-{l_{\rm T}}-{b^*}$ effects this balance. It increases the overall rate of cycling despite slowing down initial primer extension.

Further development led to the improved probe described in Fig. 2c, which utilizes an unnatural nucleotide pair (iso-dC/iso-dG, IDT) as a stopper. This tighter stopper does not require such a strong balancing weakness in the primer. A simple phosphorothioate bond between the last two primer nucleotides, with no ${b^*}$ domain, weakens hybridization enough to bias the system toward a closed hairpin. After optimizing the palindrome and conditions (see below and Methods), the cycling speed of this probe is ~5-fold faster than that of Fig. 2b.

To minimize secondary structure and prevent spurious binding and extension of primer ${a^*}-{b^*}$ or extension ${a^*}-{b^*}-{t^*}$, these domains utilize only three base types (A, T, and C). The short palindrome domain *p*, a component of template *t* in the APR cycle, is a necessary exception. See Supplementary Note 1 for other considerations.

### Proof of principle

Figure 3 presents the proof-of-principle experiments, demonstrating proximity-based, auto-cycling, Full-record generation. Figure 3a focuses on the proximity dependence of reactions. Lanes 1–4 each contained combinations of two probe types, made different only in spacer length for the purpose of differentiating products on gel electrophoresis, together with a universal Cy5 fluorophore-labeled primer and a polymerase. In lane 1, short and long barcode probes existed unbound in solution. The primers (*red*, in excess) were extended to short or long Half-records, respectively, but no Full-records were generated because probes were not in close proximity of each other. In lane 2, short barcode probes were held in proximity on streptavidin molecules, and the system produced short Half-records and, in turn, short Full-records, as expected. When streptavidin was prepared in separate vessels with either short or long probes, and then mixed together for reaction (lane 3), both Half-records and Full-records of short and long lengths were generated. Interaction among probes from different streptavidin molecules would have also produced Full-records of intermediate length (i.e., of long+short Half-records), but these were instead only present when short and long probes were randomly arranged on the same streptavidin molecules, as in lane 4.

Figure 3b demonstrates the autonomous cycling ability of the probes by observing product formation over time. Because primers were each labeled with a single copy of Cy5, the amount of product in each band could be quantified. Beginning with a 1000-fold abundance of primers (100 nM) with respect to probes (0.1 nM), probes free in solution generated undetectable levels of Full-record. When held together on streptavidin, however, each probe was utilized 30 times on average, with an initial record generation rate of ~1 min^−1^.

Conclusive proof that a given individual probe sequentially generates records with multiple different partner probes was provided by massively parallel DNA sequencing. Probes with a universal primer domain and random stem domains (creating barcodes unique to each probe) were bound by streptavidin and recording was performed. Figure 3c shows an example of a single probe (with Barcode *i*) generating records with three other probes (Barcodes ${j^*}$), as expected. See Supplementary Fig. 4 for mechanism and details.

### Probe reach characterization

**Fig. 4:**
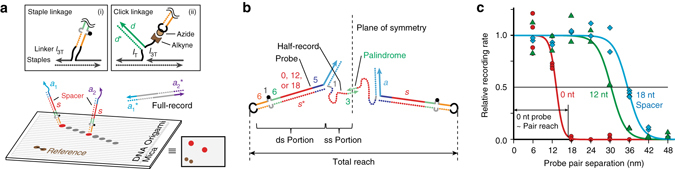
Probe reach. **a** To characterize reach, relative probe positioning was controlled precisely. Probes were attached to 2D, rectangular, twist-corrected DNA origami structures measuring ~70 by 100 nm (origami shown with all possible Fig. 4 probe positions, as well as equivalent cartoon with 4 probes; see Supplementary Fig. 5 for origami design, Supplementary Table 1 for staple sequences, and Supplementary Fig. 6 for precise probe positions). After assembly, origami were deposited randomly on mica surfaces in the presence of 12.5 mM Mg^2+^ for firm adhesion, a condition we find protects origami from degradation by polymerase (see Supplementary Note 2). Each probe was a direct extension of two origami staples (*inset* i) or covalent attachment to an intermediary oligonucleotide (*inset* ii; azide on probe loop covalently attached to alkyne on *l*_3T_−*d* intermediary via DIBO-based click chemistry and gel-purified). Separation distances were measured between origami attachment points. Records were collected from the supernatant without disturbing the underlying origami, and PCR-amplified with, for example, ${a_{1}^*}$ and ${a_{2}^*}$ primers. **b** The maximum reach across which a probe pair can make a Full-record is determined by the cumulative lengths of their components, including double-stranded probe components and single-stranded Half-record components. These are shown as rigid and flexible, respectively, corresponding to their persistence lengths of ~50 nm^38^ and ~2 nm^39^. Numbers indicate lengths in nucleotides. **c** Otherwise identical probes with spacer lengths of 0 or 12 nt (attached by staple extension), or 18 nt (covalently attached), were held in pairs separated by 6 to 48 nm in 6 nm increments (*red positions* in **a**), recorded (1 h at room temperature, 100 nM primers), and log-phase PCR-amplified (20 cycles, 500 nM primers) to gel-detectable levels. Denaturing PAGE band quantification was normalized to a constant reference pair for each well. Data for a given probe pair type were fit by least squares recursion to a simple sigmoidal curve *c*_1_/(1+Exp[*c*_2_(dist−*c*_3_)]), where *dist* represents separation distance and *c*_1_ through *c*_3_ were fit, and normalized to a maximum rate of 1. See probe sequences in Supplementary Table 2

Probe design dictates the reach of a probe pair, or probe–probe proximity within which Full-records can be produced. To characterize the rates of Full-record production as a function of probe–probe distance, pairs of probes were fixed to programmed positions on two-dimensional (2D) DNA origami^3^ structures (Fig. 4a). Three types of probes were tested, encompassing three probe spacer lengths and two attachment methods. Two types were attached as extensions of intrinsic origami staple strands (Fig. 4a, *inset* (i)), while a third was held by a click-chemistry azide–alkyne linkage to an intermediate strand (*inset* (ii)) to demonstrate how probes may be attached to arbitrary moieties. Both methods incorporated single-stranded DNA as flexible linkers at the base of each probe. Figure 4b diagrams the flexible, single-stranded and the rigid, double-stranded components contributing to the reach between two probes.

Recording reactions for each probe and separation distance were carried out separately. Many copies of a given origami structure were held flat and immobile on a mica surface, as is done for atomic force microscopy of DNA structures (see Supplementary Note 2 and Supplementary Fig. 7 for details of how this stabilizes structures against displacing polymerases). Records were then amplified by PCR, and products quantified by gel electrophoresis. To account for variation in experimental conditions, especially in the number of origami, a reference probe pair with orthogonal PCR sequences was also present on each origami type, but always at the same fixed separation distance (Fig. 4a). The rate of production was calculated in relation to this reference pair.

Figure 4c shows the relative recording rate for three probe designs, containing 0, 12, and 18 nt spacer domains *s*. Each probe pair was tested every 6 nm for separations of 6 to 48 nm, and mathematical fits to record generation rates were normalized to a common maximum. The 0, 12, and 18 nt probes all produced records at near maximum rates when pairs were closest to one another. Rates were reduced to half at 13, 20, and 25 nm, respectively, and had a maximum reach (1% of maximum recording rate) at 17, 39, and 44 nm. In absolute terms, the 0 nt spacer probe produced records at the slowest rate, approximately half as fast as longer probes.

All three maximum-reach distances correspond to those expected when DNA probes and attached Half-records are oriented in a straight chain (Fig. 4b). For the 0 nt spacer probe, the maximum expected distance is approximately twice the sum of the probe length (19 bp of double-stranded DNA, at 0.34 nm per base pair^34^, ~6.5 nm) and Half-record length (11 nt of single-stranded DNA minus 3 nt palindrome overlap, at the maximum 0.58 nm per base^34^, ~4.6 nm), totaling ~22 nm. Similarly, the 12 and 18 nt spacer probes have a maximum reach of ~44 and ~55 nm, respectively, at which recording has stopped. See Supplementary Note 3 and Supplementary Fig. 8 for estimates of reach profiles using worm-like chain models.

### Interrogation of complex geometries

**Fig. 5:**
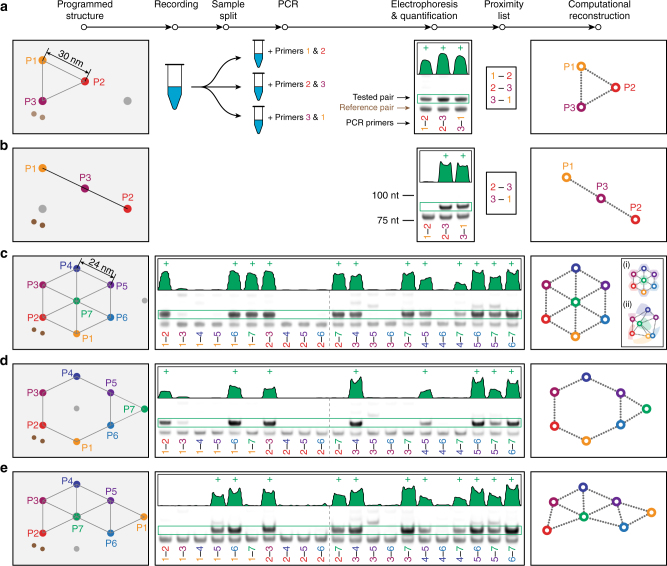
Complex geometry interrogation. **a** The same rectangular origami base used in Fig. 4b was adapted to hold three probes, of 18 nt spacer design, at 30 nm spacing (see Supplementary Fig. 6). In the same conditions as in Fig. 4, probes were first placed in a triangular arrangement and recording was carried out. The sample was split, records PCR-amplified with separate pairs of probe primers (and reference primers), and products separated by gel electrophoresis and quantified (*green plot profiles*, marked (+) when judged to qualify for the proximity list). As expected, all three probe pairs yielded records, suggesting a triangular shape in reconstruction. **b** When those same probes were attached in a line, with P1 and P2 out of reach from one another, only two record types were amplified. This suggests a relatively linear arrangement. **c** More complex, 7-probe geometries were also interrogated, this time with 24 nm hexagonal grid spacing and 12 nt spacer probes. The full set of 21 primer pair amplifications yielded the gel shown, with 12 pairs in proximity. Computational reconstruction yielded the same hexagonal pattern. *Insets* show positional precision of any single point, with respect to others shown, as *shaded region* that satisfies this gel. *Inset* (i) shows the precision for computed hex pattern, while *inset* (ii) shows the precision for a random pattern that also satisfies gel result. **d** When center probe P7 was moved outside the hexagon, APR yielded the 8 expected proximities and correct reconstruction. **e** Similarly, when P1 was moved, the 11 expected proximities and correct reconstruction resulted. Throughout, computational reconstruction images were reflected and rotated, but not distorted, for best comparison to the programmed structure diagrams. See probe sequences in Supplementary Table 2 and full gels in Supplementary Fig. 9. All reference bands in the figure correspond to lengths indicated in **b**

APR can be used to gently interrogate extensive proximity relationships within a homogeneous sample of structures, enabling reconstruction of overall geometry from PCR-based assays (Fig. 5). The longest probe tested in Fig. 4c, with an 18 nt spacer (*s*), was first used to determine the relative proximities of three targets. Three probes, programmed with different primer binding sequences (*a*) and therefore generating records with unique ends (e.g., ends $a_{\rm{i}}^{\rm{*}}$ and *a*_j_ in Fig. 2a), were again fixed by 2D origami in a triangular configuration with 30 nm probe separation (Fig. 5a). This placed each probe within the reach of the other two. A recording was performed, as before, and the sample was split into three volumes, reflecting the number of potential pairwise probe combinations. Each subsample was combined with a different pair of PCR primers, and records, if any, were amplified. Gel electrophoresis and band quantification indicated if records were produced, and a graph depicting relative positioning was computationally reconstructed. As expected, the triangular arrangement yielded all three record types, suggesting that very geometry. In contrast, when the same probes were placed in a straight line (Fig. 5b), only two record types were produced, as the distance between P1 and P2 was beyond probe reach.

More complex, 7-point geometries were then tested, each using shorter, 12 nt spacer (*s*) probes arranged on a smaller, 24 nm hexagonal pattern (Fig. 5c–e). This made nonadjacent probes at least 42 nm apart, beyond the reach of these probe pairs (≲39 nm). Each 7-probe geometry required testing of 21 (6 + 5 + 4 + 3 + 2 + 1) possible probe pairings, yielding one of 2^21^ > 2 × 10^6^ possible binary combinations, any potentially valid. As in Fig. 5a, records were produced from these origami, split, and amplified by PCR in the 21 possible primer pairings. The hexagonal geometry of Fig. 5c yielded 12 of 21 positive proximity pairs, as seen by gel electrophoresis, in addition to the 21 reference pairs that served as positive PCR controls. Simple graphing of these 12 pairs (Wolfram Mathematica, GraphPlot function, see Supplementary Note 4) resulted in the correct geometry.

The exceptional fidelity of reconstruction in this case is due to a combination of experimental and reconstruction details. The graphing algorithm used here is based on a physical model (spring-electrical) that separates the final probe positions relatively uniformly, and is therefore particularly congruent with the regular hexagonal organization of the origami probes. A sense of the precision of this reconstruction is shown in Fig. 5c, *inset* (i). Given the positions shown for any six probes, the seventh probe may be anywhere within the shaded region and still satisfy the gel data shown. In addition, a series of graphs with random probe positions were generated and tested for compatibility with these same data. One compatible example is shown in the Fig. 5c *inset* (ii), again with *shaded regions* showing the precision of a given probe position. See Supplementary Fig. 10 for more examples.

Two further examples are demonstrated. Figure 5d, where the center probe was moved to the outside, yielded the eight correct proximity pairings and geometry. Figure 5e, where the lower probe was moved to the outside, yielded the 11 correct proximities and geometry.

Note that the relative quantity of Full-records created should not be taken as a measure of relative distance between probes, but only that the appearance of records indicates that probes were within reach (proximity); the nonlinear record generation rate as a function of distance (Fig. 4c), variable probe attachment to target, and possible sequence dependence make distance approximation from precise record count unreliable. Instead, further refinement of target–target distances may be performed by sequential experiments with probes of different reach. For example, a pair of 12 nt spacer probes may produce records, indicating targets closer than 36 nm, while a pair of 0 nt spacer probes at those same positions do not, indicating those same targets are also farther than 18 nm. Alternatively, a single experiment with a mixture of probes with unique primers and variable reach could be used. See Supplementary Fig. 11 for examples of how reconstruction precision could be further refined with this strategy.

### Resampling of a state-changing system

**Fig. 6:**
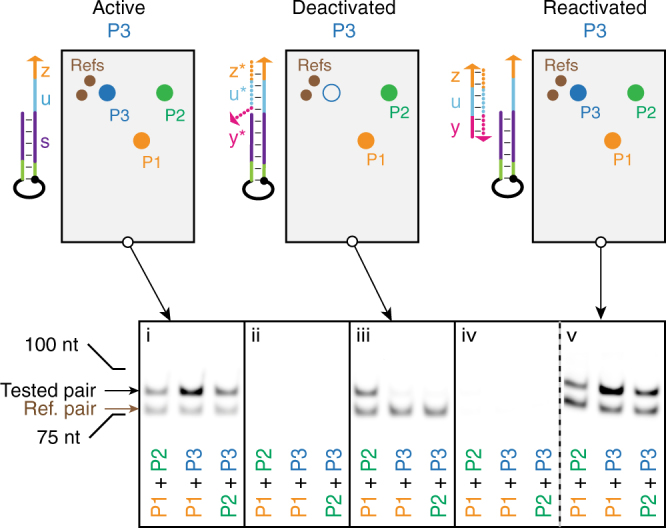
State change. The same origami probe triangle was used as in Fig. 5a, but probe P3 could be deactivated. (see Supplementary Fig. 6 for precise probe positions.) PCR amplification and denaturing PAGE of recording from active probe (i), after intermediate wash (ii), from deactivated probe (iii), after second intermediate wash (iv), and from reactivated probe (v). The intermediate wash results demonstrate that there were no leftover records. A single set of origami was used for all steps and resampled. Origami and PAGE otherwise treated as in Fig. 5. See probe sequences in Supplementary Table 2 and full gels in Supplementary Fig. 12

An advantage of nondestructive recording is that the same probes can be repeatedly sampled, even in a system undergoing changes in its state. The origami-based triangular arrangement of probes in Fig. 5a was again constructed (Fig. 6), with a mechanism for inactivating probe P3 by shielding its primer binding site (*u*−*z*) with a blocker strand (${z^*}-{u^*}-{y^*}$), as well as for subsequently reactivating P3 by removing the blocker with strand *y*−*u*−*z*. As such, the same tri-target system can be made to cycle between two distinct states, with P3 initially active (stage i, Fig. 6), then inactive (stage iii), and finally active again (stage v).

We applied APR to interrogate this state-changing system. At each sampling point, the supernatant was split, PCR amplified, and observed by gel electrophoresis. First, the triangular arrangement was recorded (with primer ${u^*}$ on P3) and analyzed, indicating the colocalization of three probes as expected (Fig. 6, i). After a wash with buffer, the supernatant showed no residual records (Fig. 6, ii). Then, by applying inextensible (inverted dT at 3′ end to prevent extension) P3 primer blocker ${z^*}-{u^*}-{y^*}$, any leftover P3 primers and Half-records were displaced and probe P3 was made unable to bind primer, rendering it deactivated. Recording then indicated colocalization of only P1 and P2 (Fig. 6, iii). To sample a third state, the same origami were again washed and shown record-free (Fig. 6, iv). Probe P3 was then reactivated by addition of strand *y*−*u*−*z*, which removed the primer blocker. Recording and analysis once again indicated the full triangular arrangement (Fig. 6, v), completing three correct samplings of the same targets.

## Discussion

Given that structure dictates function in synthetic and natural nanostructures, we sought to record nanoscale proximity and organization in as precise and complete manner as possible. To this end, we have developed a biochemical technique termed APR. The two key mechanisms requiring development were a nondestructive, continuous (i.e., auto-cycling) method for copying DNA strands from probes, and a method for pairing these strands only in the presence of probe proximity (proximity recording). The first was solved by the development of the CRH (also recently suggested elsewhere^35^), which allows a probe-specific primer to be extended into a closed hairpin using the energy in deoxynucleoside triphosphates (dNTPs), and then isothermally releases the template by re-closing the hairpin via the strand displacement process. The second, proximity requirement was enforced by encoding a short palindromic site at the 3′ end of the extension. The extended primers represent Half-records that must pair by their palindromes with other nearby Half-records before they can extend over each other, through their primer domains, and be released into solution as Full-records of proximity. Subsequent identification of these record sequences reveals the pattern of proximity.

The APR method shares a superficial resemblance to PLA and PEA techniques in that all three record pairwise interactions in DNA. A key difference is that because APR probes are not consumed in detecting each proximity, many pairwise recordings reflecting the underlying equilibrium conformation can be made from each probe. We have taken advantage of this to demonstrate the detection and reconstruction of complex geometries and the resampling of the same molecular system in different states, all with simple PCR and gel-based readouts. The geometry reconstructions placed 7 probes in the correct relative position, a 1-in-2 × 10^6^ event by chance alone. Resampling allowed the single sample to be recorded in three different arrangements. Neither, to our knowledge, have been demonstrated with proximity ligation or other biochemical techniques. While pairwise ligation of many copies of a target might be expected to yield the same geometry results, in our own previous attempts ligation was often detected between probes that were implausibly far apart. We hypothesize that ligation operates as a latch that permanently connects even rare pairings distorted under thermal motion. These improper ligations distort the underlying geometry and make further pairings inaccurate as well. Conversely, APR makes only transient connections between probes, allowing the underlying proximities to reflect the desired equilibrium state.

No ensemble experiment detecting pairwise proximities can differentiate between a complex structure and separate pairings that overlap to form that structure. Because APR generates ~30 records per probe, providing sensitivity to most or all neighboring connections, it suggests the challenging but exciting prospect of single-molecule analysis. Random probe barcodes could be used to uniquely identify each of, for example, 10^6^ molecular targets, and massively parallel next-generation sequencing could read out every recorded proximity on a per-molecule basis. Specificity against chance (non-repeating) interactions could be enforced by neglecting any proximity read found only once^36^. Such a single-molecule capacity may enable, for example, an assay that counts any number of multiplexed protein types in cell lysates (by applying antibodies to two separate epitopes of each protein type), measurement of molecular interaction stoichiometry and turnover, and of course single-molecule network connectivity that differentiates complexes from separate components. Alternatively, non sequencing-dependent detection of a soluble, low-concentration protein could suppress false positives by requiring two interactions between the same probe pair to create a single record. We are actively pursuing several of these capabilities.

## Methods

### Probes

Probes were ordered from IDT with possible modifications of internal biotin (/iBiodT/), internal azide (/iAzideN/), internal Spacer 9 (/iSp9/), internal iso-dC/dG pairs (/iMe-isodC/ and /iisodG/), or phosphorothioate bonds (denoted by “$^*$” between nucleotides). See Supplementary Fig. 2 and Supplementary Tables 1 and 2 for probe sequences. Strands were ordered at 100 or 250 nmol scale, with high-performance liquid chromatography (HPLC) purification.

Some probes were ordered with an internal biotin modification in the center of the 5 nt loop domain, in which case probes were held in proximity with streptavidin, as indicated in Fig. 3 and Supplementary Fig. 2. Probes were attached to streptavidin (New England Biolabs (NEB)) in a 20 μl reaction containing 25 nM streptavidin (NEB) and 140 nM probe (for a total of more than 4:1 probe/streptavidin to ensure streptavidin saturation), held at 37 °C for 1 h.

Other probes were covalently linked to alkyne-modified strands by click chemistry. Azide-modified raw probes were ordered directly from IDT and dibenzocyclooctyne (DBCO) group modified linker strands were purchased from Boston Open Labs. Both DNA strands were HPLC purified by the manufacturers. A volume of 9 μl of 1 mM DBCO-linker strands and 1 μl of 10× phosphate-buffered saline (pH 7.4) buffer were added to 20 μl of water containing 180 pmol azide-modified probes and incubated at room temperature for 12 h. The linked probes were purified by 8% denaturing polyacrylamide gel and quantified by NanoDrop UV–Vis Spectrophotometer (Thermo Scientific).

For random barcode probes, a chemically-synthesized probe was purchased with a 5′ extension representing the complement of the primer-binding portion. Several thymine nucleotides were replaced with uracil in this region. The probe contained random nucleotides for the competitor side of the probe (as in Suppl﻿ementary Fig. 2b) and ended with the 3′ palindromic domain. This domain was subsequently extended with Bst large fragment polymerase (NEB M0275), followed by USER-mediated cleavage (Uracil-Specific Excision Reagent, NEB, M5505) of the 5′ uracil-containing domains and polyacrylamide gel purification.

### NUPACK simulations

Expected reaction products at thermodynamic equilibrium were predicted using NUPACK^32^ web-based software (nupack.org), analysis option. Specified options were DNA nucleic acid type, 37 °C temperature, 10 nM reactants, with maximum complex size of two. Advanced options 0.05 mM Na^+^, 0.002 mM Mg^2+^, and all dangle treatments. Pair probability results were downloaded in text form and replotted using custom software (Wolfram Mathematica). See Supplementary Fig. 1.

### APR recording

Recording in solution for most experiments was carried out as follows. Probe strands were bound to streptavidin, as above. A mixture of 100 μM each dNTP (NEB), 1× ThemoPol Buffer (NEB, N0447), and 0.8 U/μl Bst large fragment polymerase, 200–400 nM Cy5-labeled primer (IDT), and probe/streptavidin mix (per above) at 10 nM final streptavidin concentration was prepared at 4 °C at a volume of 10 μl per reaction. The temperature was raised to 37 °C for 120 min (or other reaction time), followed by 80 °C heat inactivation for 20 min. The fastest reliable recording conditions found (specifically for newly optimized probe and conditions of Fig. 3b) utilize the iso-dC/dG probes, 100 nM primer (with phosphorothioate bond, IDT), 1× Bsm buffer, 1.2 U/μl total Bsm (Thermo Fisher, EP0691), 150 μM dNTPs, and an additional 4 mM MgSO_4_.

For recording reactions on origami, flow chambers were created by attaching a freshly cleaved mica (Ted Pella) piece to a channel slide system (ibidi, Sticky-Slide VI0.4, Cat. No. 80608), yielding a 30 μl chamber volume. After washing the chamber three times with 60 μl of TAE/Mg buffer by adding the buffer to one reservoir (inlet) and subsequently taking out the same volume from the other side (outlet), a 30 μl solution containing origami at 50 pM concentration was introduced to the chamber. (In the first washing round, ~30 μl of buffer occupies the chamber and only ~30 μl of extra buffer comes out.) After 30 min of incubation, unbound origami and extra staple strands were removed by washing three times with 60 μl of fresh TAE/Mg buffer (40 mM Tris acetate, 1 mM EDTA buffer supplemented with 12.5 mM magnesium acetate) and three times with 60 μl of 1× ThermoPol buffer (NEB, Cat. No. B9004S, 10× ; 1× contains 20 mM Tris-HCl, 10 mM (NH_4_)_2_SO_4_, 10 mM KCl, 2 mM MgSO_4_, 0.1% Triton X-100). To the chamber, a 40 μl solution containing 0.8 units/μl of Bst polymerase (NEB, Cat. No. M0275S), 100 μM dNTP (NEB, Cat. No. N0447S), and the relevant primer mixes typically at 100 nM each, in 1× ThermoPol reaction buffer, was added and incubated for 1 h at room temperature. After the recording reaction, the supernatant solution containing produced records was collected and the polymerase was heat inactivated by incubating the solution at 80 °C for 20 min. For samples used in the geometry studies (three-point and hexagonal grid patterns) and the state change study, before quenching the reaction, extra recording primers contained in the product solution were removed by mixing the solution with Exonuclease I (NEB, Cat. No. M0293S) at 9:1 volume ratio and incubating for 20 min at 37 °C. This minimizes any false-positive PCR amplification by the recording primers.

State change recordings of Fig. 6 were carried out following the standard origami procedure described just above, except that the product solutions were collected after first adding 40 μl of buffer into the inlet, to prevent drying of the chamber between rounds. After the first and second state recording rounds, the chamber was washed six times with 1× ThermoPol buffer before the wash samples were collected, followed by three times with TAE/Mg. For deactivating or reactivating the probe P3, a 40 μl solution containing the deactivator strand at 10 μM or the reactivator strand at 0.1 μM respectively in TAE/Mg was added and incubated for 30 min at room temperature, after which the chamber was washed four times with TAE/Mg and four times with 1× ThermoPol buffer before each recording reaction.

For the 7-point hexagonal grid geometry recordings of Fig. 5, the experimental condition is similar to the ones for stage change recording except that the annealing temperature for PCR amplification was to 52 °C, with 200 nM of each primer sequence used in the PCR mixture.

### Electrophoresis

Gels (8 × 8 cm) for denaturing polyacrylamide gel electrophoresis (PAGE) were cast in house at 8% acrylamide (J.T.Baker), with 7 M urea (Sigma) and 1× TAE (40 mM Tris, 20 mM acetic acid, 1 mM EDTA) buffer (GrowCells.com), in plastic cassettes (Life Technologies), and run for 30–35 min at 200 V and 65 °C in 1× TAE buffer. Gels were then removed from cassettes, stained in 1× SybrGold (Life Technologies) for 15 min, and imaged with a Typhoon scanner (General Electric). All ladders shown are 10–300 nt (Thermo Scientific, Cat. No. SM1213).

### DNA origami preparation

The design and folding protocols of the 2D rectangular origami were adapted from the original rectangle^3^ and the twist-corrected version^13, 37^. Single-stranded M13mp18 DNA (scaffold strand) was purchased from Bayou Biolabs (Catalog P-107) and staple strands were obtained from IDT. Staple strands were purchased and used unpurified, except the staples with probe extension at the 5′ end of the staples, which were purchased PAGE-purified. Scaffold and staples were mixed together at target concentrations of 5 and 40 nM, respectively, in TAE buffer with 12.5 mM magnesium acetate. For origami with click-coupled probes, the probe strands were added at 1.2-fold the concentration of the staple strands containing the anchor. For origami folding, the mixtures were kept at 90 °C for 5 min and annealed from 90 to 60 °C over the course of 30 min, then from 60 to 45 °C over the course of 90 min, and finally from 45 to 25 °C over the course of 20 min. Folded origami solutions were used without further purification except where otherwise stated. See Supplementary Fig. 5 for geometry and layout and Supplementary Table 1 for staple sequences.

### Record PCR amplification

PCR amplification utilized 1× VentR (exo-) polymerase in 1× ThermoPol Buffer (NEB), for 20 cycles, in 20 μl reactions. Primers were initially present at 0.5 μM, and dNTP mix (NEB) at 100 μM each. Temperature cycling included a 95 °C melt for 2 min, 20 cycles (varies) of 95 °C for 20 s denaturing, 56 °C for 45 s annealing, 72 °C for 15 s extension, and a final 72 °C extension for 3 min.

### Data availability

The data that support the findings of this study are available from the authors on reasonable request.

## Electronic supplementary material

Supplementary Information
