# Supplementary material for: A DNA nanoscope via auto-cycling proximity recording
Source: Nat Commun. 2017 Sep 25;8:696. doi: 10.1038/s41467-017-00542-3 (PMC5612940; doi:10.1038/s41467-017-00542-3)
Supplement: Supplementary file 1 — Supplementary Information [file 41467_2017_542_MOESM1_ESM.pdf]

File Name: Supplementary Information
Description: Supplementary Figures, Supplementary Tables, Supplementary Notes and Supplementary References

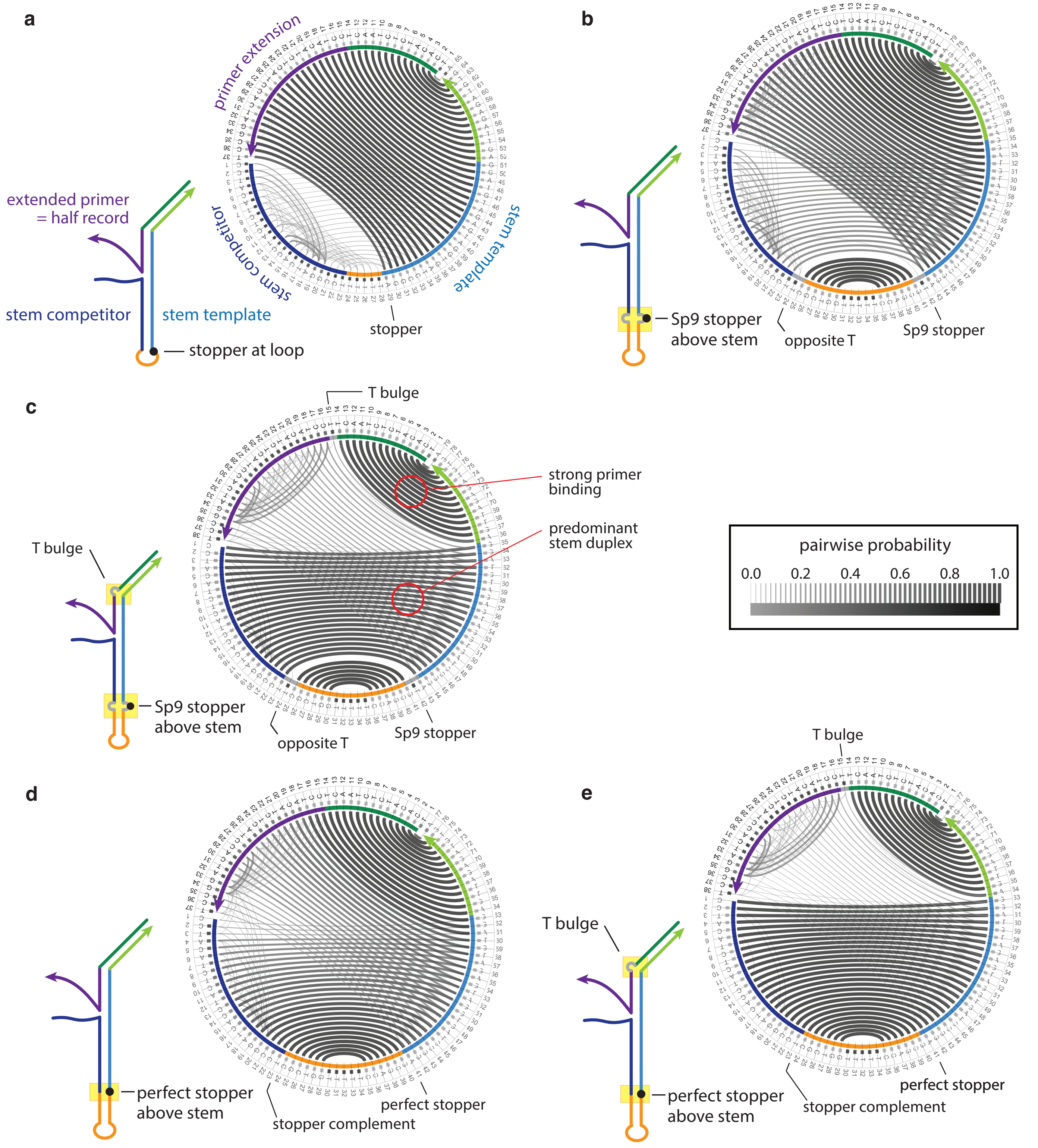

Supplementary Figure 1: **Probe design and thermodynamically-calculated performance.** Essential functional domains of the probe are shown in the inset probe diagrams. All pairwise probabilities of the extended primer - probe ordered complex calculated using nupack.org[1] web server using default DNA options plus: Temperature: 37C, Number of strand species: 2, Maximum complex size: 2, Strand species as in Supplementary Table 2, Dangle treatment: All, Na^{+}: 0.05 M, Mg^{2+}: 0.002 M. Data were downloaded as a pair probability text file and re-plotted with custom Wolfram Mathematica code (to be published elsewhere). A single T nucleotide is used to substitute for IDT “Sp9”, as there are no relevant thermodynamic data. Stem template is arbitrary 3-letter code (see Supplementary Figure 2 and Supplementary Table 1). Strands are arranged around a circle, with the darkness and width of lines connecting the nucleotides indicating higher probability of association, per key. (**a**) A stopper modification ends polymerization at a small hairpin loop, yielding a primer predominantly bound to the stem template. (**b**) Some improvement in performance is achieved by adding a small stem below the stopper. (**c**) In one commonly used probe, an Sp9 stopper (IDT) is paired with a primer T bulge nucleotide, making the stem predominantly closed. (**d**) A “perfect” stopper is represented by a C-G pair, modeling the iso-dC/dG (IDT) pair used in the highest performing probe presented in the manuscript. (**e**) Even then, there is a benefit to a T bulge or other primer-weakening modification. See Supplementary Note 1.

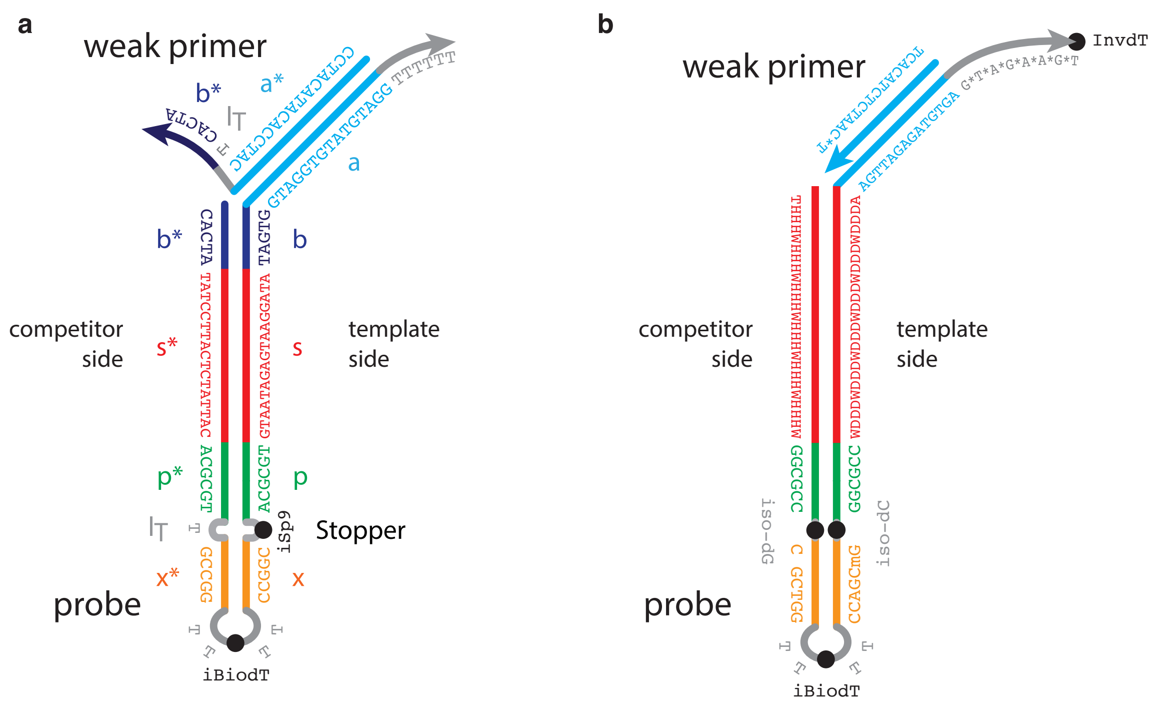

c

```
probes:                            ------- competitor side -------- ------- stopper and biotin --------- ------- template side ---------- – primer site –
Main Text Fig. 3a (prox., short):   CACTATAT                  ACGCGT T  GCCGG TT/iBiodT/TT CCGGC   /iSp9/ ACGCGT                  ATATAGTG   GTAGGTGTATGTAGGT...
Main Text Fig. 3a (prox., long):    CACTATATCCTTACTCTATTAC    ACGCGT T  GCCGG TT/iBiodT/TT CCGGC   /iSp9/ ACGCGT GTAATAGAGTAAGGATATAGTG   GTAGGTGTATGTAGGT...
Main Text Fig. 3b (t series):      THHHWHHHWHHHWHHHWHHHWHHHW GGCGCC ˇ CGCTGG TT/iBiodT/TT CCAGCmG  ˇ     GGCGCC WDDDWDDDWDDDWDDDWDDDWDDDA AGTTAGAGATGTGA...
                                                                /iisodG/                          /iMe-isodC/                   → ...G*T*A*G*A*A*G*T /3InvdT/

primers:
Main Text Fig. 3a:                 /5Cy5/ TT CCTACATACACCTAC T CACTA
Main Text Fig. 3b:                 /5cy5/ TCACATCTCTAAC*T
```

Supplementary Figure 2: **Probe sequence details**. (**a**) Probe detail figure, with T-bulge primer attached but not yet extended. Superimposed sequences correspond to Main Text Figure 3a, long probe. Domain labels correspond to Main Text Figure 2a. (**b**) Probe detail of the highest-performing probe, correspond to Main Text Figure 3b. Changed include deletion of domains l_T and b, a new phosphorothioate bond at primer end (...AAC*T), stopper replacement with an iso-dC and iso-dG pair, and a better probe 3' end protector, InvdT (= idT = inverted dT). (**c**) A list of sequences for all three probes used in Main Text Figure 3a and b, as well as the primer sequences. Sequences are written in IDT format, with /iBiodT/ as code for a biotinylated T nucleotide, /iSp9/ as code for a non-DNA spacer of length ~1 nt, inserted internally, /iisodG/ and /iMe-isodC/ for the Iso-dC/dG pair, an "m" preceding a nucleotide to denote a 2'O-Me RNA base (backup stopper), and an * to denote a phosphorothioate link. IUPAC code "D" represents a random nucleotide of A, G, or T identity, "H" represents A, T, or C, and "W" represents A or T. The probe used in Main Text Figure 3a is used predominantly in this paper, including main text Figs. 4c, 5, and 6, in conjunction with the T-bulge primer. Later development of a higher-performance probe, that of Main Text Figure 3b and used in conjunction with a phosphorothioate primer, is also shown.

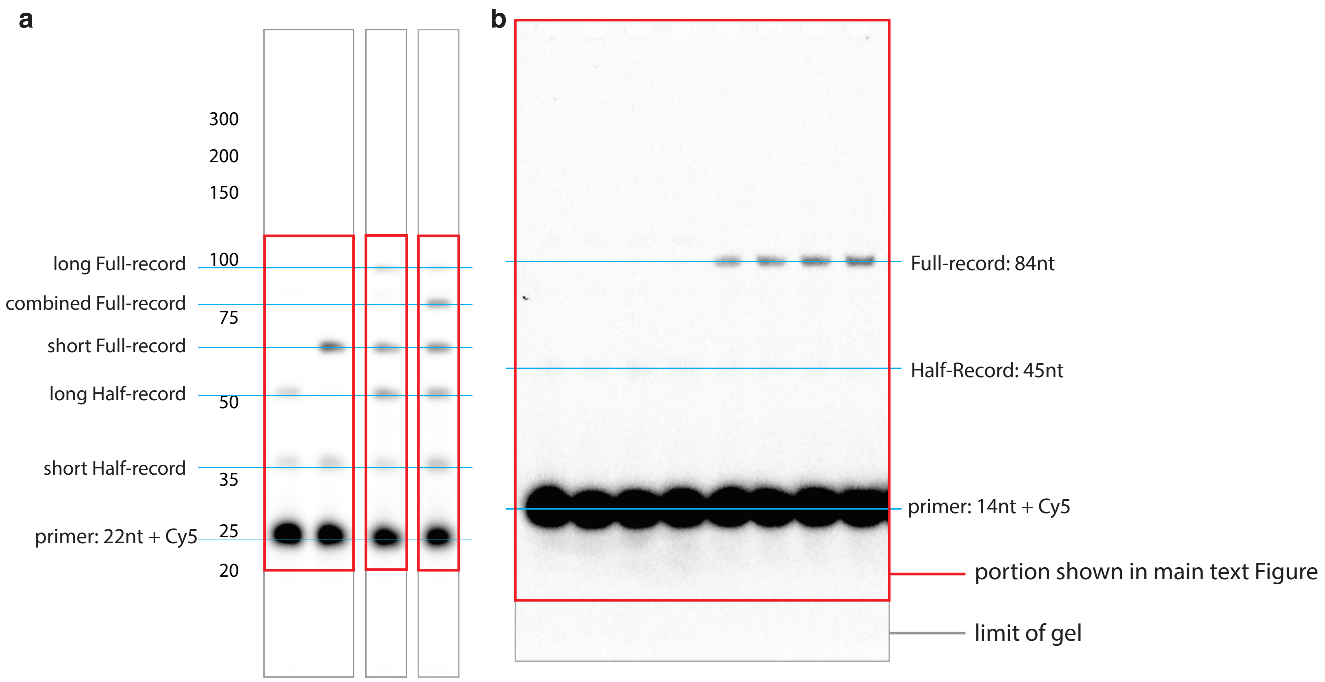

Supplementary Figure 3: **Full, un-cropped gel lanes for Main Text Figure 3**. The gel for Main Text Figure 3a appears in (a), and that for Main Text Figure 3b in (b). Only the Cy5 channel (i.e., primers and extensions) is shown. Ladder position (ladder not shown), lanes, and main text figure cropping as noted.

a

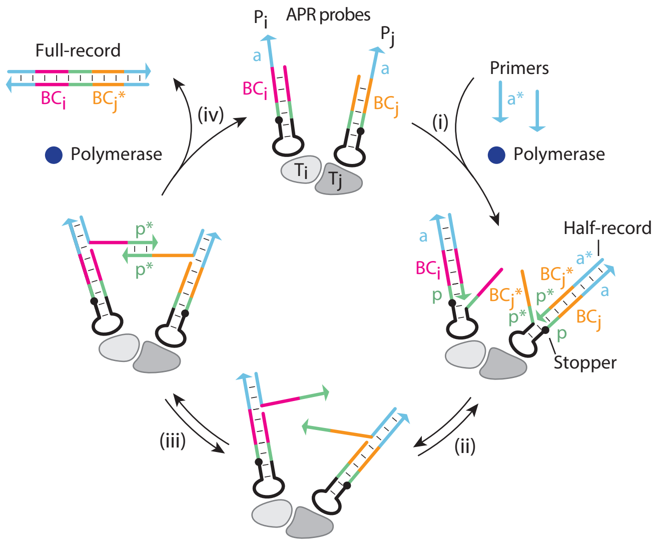

b

```
         -------- competitor side ------ ------- stopper and biotin -------- -------- template side -------- -- primer bind site -
probe:   CCTAC HHHHHHHHHHHHHHHHHH AGGCCT T CGCTGG TT/iBiodT/TT CCAGCG /iSp9/ AGGCCT DDDDDDDDDDDDDDDDDD GTAGG AGTTAGAGATGTGA TTTTTT
               barcode complement                                                    - random barcode -

primer:  TCACATCTCTAACT T CCTAC
```

c

```
              Universal primer              Barcode i       Palindrome p     Barcode j *          Universal primer *
5'                                                                                                                  3'
  ...CAA ATCACATCTCTAACTTCCTAC AACAAATACAAATATTCT AGGCCT ATAATATAAGTATTGGTA GTAGGAAATTAGAGATGTGAA TAG...
  ...GTT TAGTGTAGAGATTGAAGGATG TTGTTTATGTTTATAAGA TCCGGA TATTATATTCATAACCAT CATCCTTTAATCTCTACACTT ATC...
3'                                                                                                                  5'
5'                                                                                                                  3'
  ...CTA ATCACATCTCTAACTTCCTAC AATTCAACAAATTAATCA AGGCCT AGAATATTTGTATTTGTT GTAGGAAGTTAGAGATGTGAA ATG...
  ...GAT TAGTGTAGAGATTGAAGGATG TTAAGTTGTTTAATTAGT TCCGGA TCTTATAAACATAAACAA CATCCTTCAATCTCTACACTT TAC...
3'                                                                                                                  5'
5'                                                                                                                  3'
  ...CAA ATCACATCTCTAACTTCCTAC TATTCTACTTCTATGCAT AGGCCT AGAATATTTGTATTTGTT GTAGGAAGTTAGAGATGTGAA ATG...
  ...GTT TAGTGTAGAGATTGAAGGATG ATAAGATGAAGATACGTA TCCGGA TCTTATAAACATAAACAA CATCCTTCAATCTCTACACTT TAC...
3'                                                                                                                  5'
```

Supplementary Figure 4: **Parallel sequencing shows multiple partnerships - Expansion of data in Main Text Figure 3c** Massively parallel sequencing of Full-records from four streptavidin-bound probes (as in Main Text Figure 3a, inset) with unique spacer sequences (i.e., barcodes) demonstrates that a single probe can make partnerships and Full-records with multiple other probes. Each probe in such a cluster of 4 can potentially form Full-records with three different partners. (**a**) The essential mechanism is the same as that of Main Text Figure 2a, except that spacers have been replaced with barcodes unique to each target and that primers are all the same ("Universal"). (**b**) The probes used in this experiment had the universal primer site but randomly-generated barcodes ("H" stands for any of A, C, or T, randomly inserted in each probe). The probe is ordered with the 18-nucleotide poly-H sequence and then manufactured (extended) in house to contain its complement (denoted as poly-"D") as shown. Because an experiment utilized less than $5E5$ probes from a pool of 3^{18} ~$4E8$ possible barcodes, it is very unlikely that any given barcode was used twice. Also shown is the universal primer sequence. (**c**) Full-records were generated and sequencing adapters ligated (see below). The sample was sequenced (Illumina MiSeq), a barcode (AACAAATACAAATATTCT) was chosen from the resulting text (fastq) file, and the file was searched for records with this sequence or its complement (i.e., those strands resulting from when a universal primer was extended first on another probe). Three such Full-records are shown, with the common barcode in bold print. The three (different) paired barcodes are highlighted in yellow, and show that probes not only make multiple Full-records (consistent with Main Text Figure 3b), but that records are made with multiple different probes. Expected primer and palindrome components were also present, as shown. (**Method of sequencing**) Recording samples (i.e., containing Full-records, leftover primers, etc.) were prepared as above. The sample was immediately subjected to 15 cycles of PCR amplification (conditions below), followed by Qiaquick spin column purification and elution in EB Buffer (Qiagen). The sample was re-annealed from 80°C to 50°C over 45 min to create double-stranded ends, and subjected to Klenow fragment polymerase in NEB2 buffer (NEB) to add an "A" nucleotide to each 3' end. Modified Illumina adapter sequences were ligated with T4 DNA ligase in T4 ligase buffer (NEB) over 1 hour, and the sample was purified by denaturing PAGE (see main text methods), gel stained with SybrGold (Life Technologies), and appropriate bands were cut out under Typhoon scanner (General Electric) observation. A final 8 cycles of PCR with adapter sequence primers was performed, and sequencing was performed at a core facility with an Illumina MiSeq sequencer (v3 chemistry, 150 nt paired end reads). Fastq files were analyzed with Wolfram Mathematica.

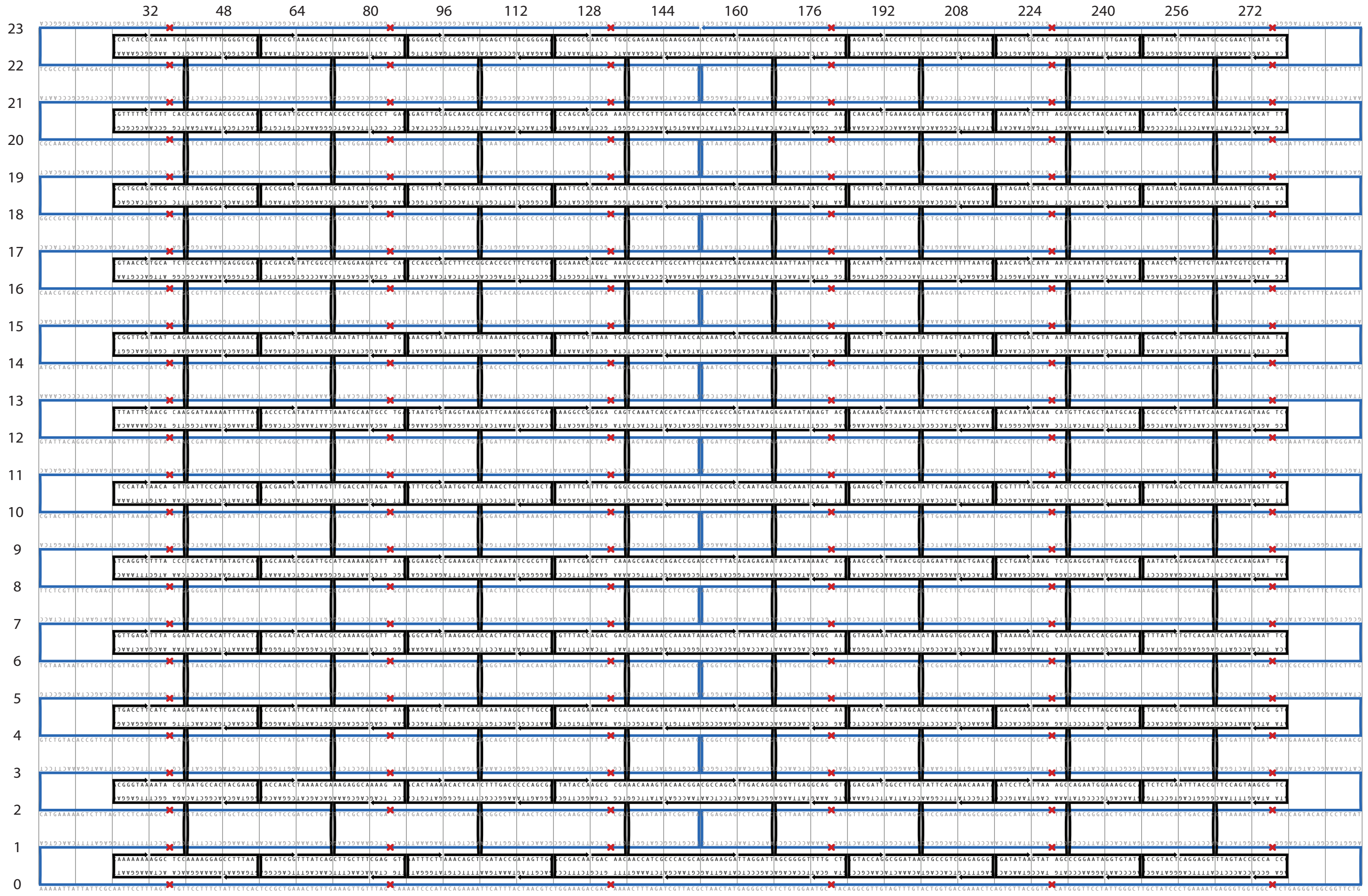

Supplementary Figure 5: **Origami routing**. Sequence diagram of the rectangular DNA origami used in this study. The blue strand represents the standard M13 scaffold strand (Bayou Biolabs) and the black strands depict the staple strands. Red "x" marks indicate the positions of "skips" for flattening (correcting for the global twist of) the origami. Sequences are superimposed; magnify pdf file to view alignment.

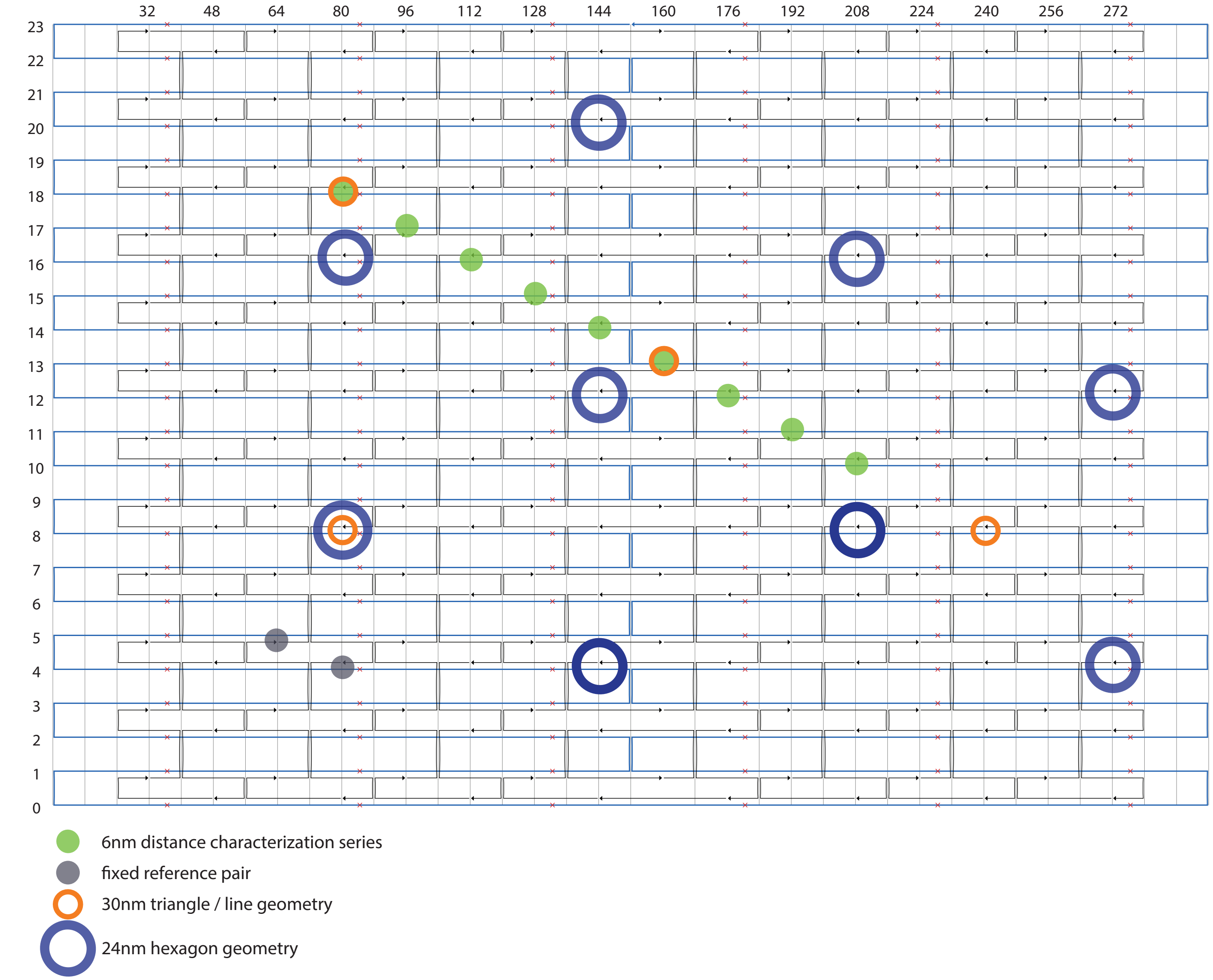

Supplementary Figure 6: **Origami probe positions**. Each filled or open circle represents the position of a probe in a main text experiment, and is color-coded as described. Light green: 9 distance series test positions of Main Text Figure 4. For staple-extension-based connections, the staple strand whose 3' end falls at the marked position and the staple whose 5' end falls at the marked position were modified to hold the corresponding probe sequences. For click-chemistry-anchored probes, the anchor sequences were attached to the 5' end of the staple at the position. See the sequence list in Supplementary Table 1 for details. Gray: 2 reference probes for Main Text Figures 4, 5, and 6. Orange: 4, 30 nm-spaced triangle-versus-line test positions for Main Text Figures 5 and 6. Blue: 9, 24 nm-spaced hexagonal grid positions for Main Text Figure 5. The coordinates (helix and base number) in this diagram match those in the routing diagram (Supplementary Figure 5) and sequence list (Supplementary Table 1). See Supplementary Table 2 for corresponding probes and other sequences.

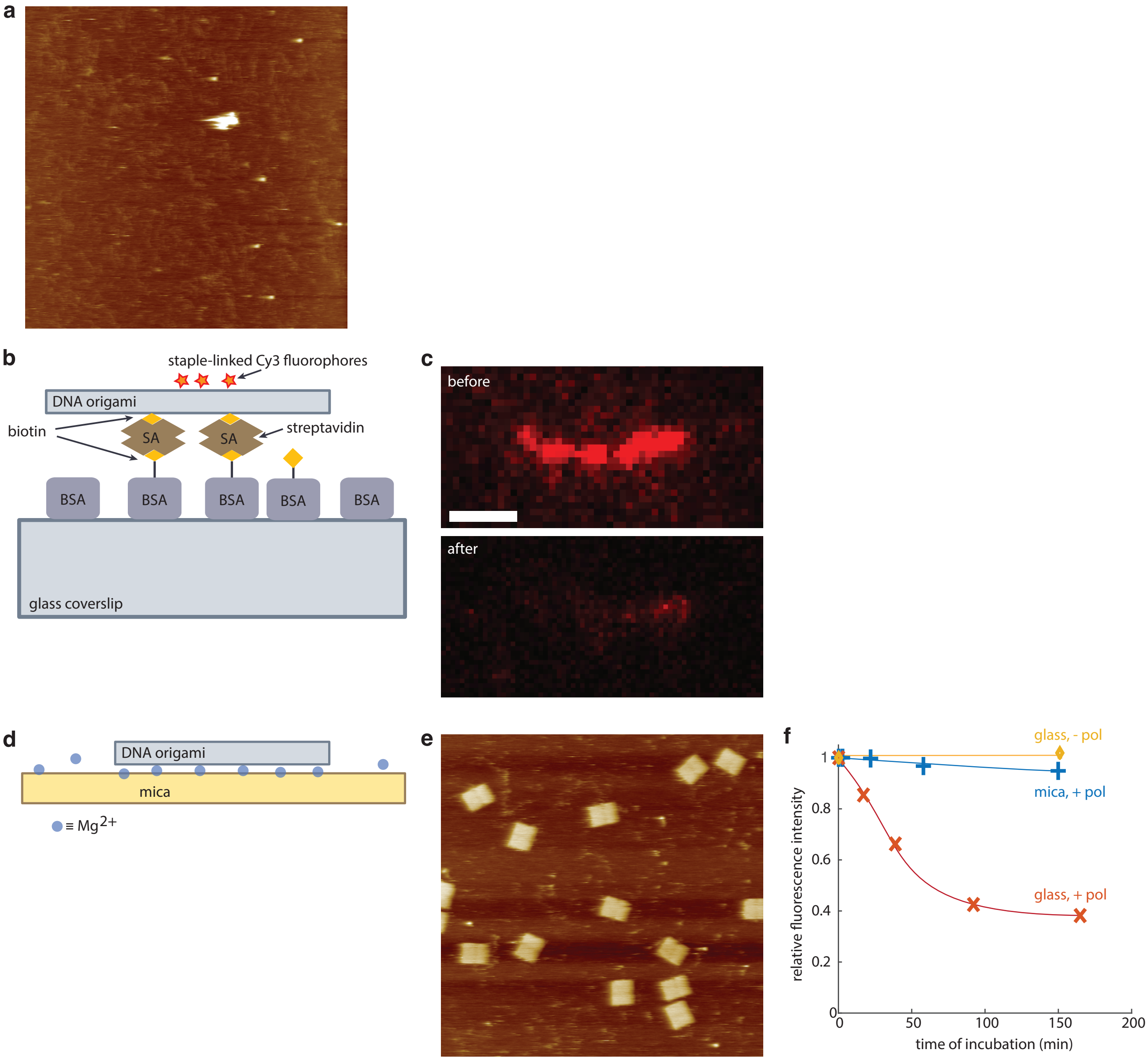

Supplementary Figure 7: **Mica-bound origami is resistant to damage by a displacing polymerase**. (**a**) An AFM image indicates that DNA origami in solution is destroyed upon incubation with displacing polymerase (Bst) and dNTP. Incubation was in 1x ThermoPol buffer at 37°C for 1 hour. Image is 1 μm by 1 μm. (**b**) Our standard method of fixing origami to the surface for visualization is to incorporate biotinylated DNA staples in the origami. These biotin bind streptavidin, in turn bound to the surface through biotinylated Bovine Serum Albumin (BSA). The BSA is also used to passivate the surface. (**c**) Employing other, Cy3-labeled staple strands in origami allows them to be visualized by Total Internal Reflection Fluorescence (TIRF) microscopy (before), but even held on surface the Bst destroys and releases the Cy3 over the course of ~1.5 hr (after). Staple strands on the edges of these origami create blunt ends and allow stacking and formation of linear chains to help identify the structures under the microscope. Scale bar is 1 μm. (**d**) DNA origami may also be held on a mica surface with Mg^{2+} cations. (**e**) An AFM image (1 μm by 1 μm) shows that origami held to the mica surface were not harmed by a 2.5 hr Bst incubation at room temperature. (**f**) Similarly, and in contrast to those held on glass surfaces, Cy3-labeled strands held on origami were not destroyed while on mica. The relative fluorescence intensity was measured as the average fluorescence in pixels containing signal (above a certain threshold, determined for each image to differentiate signal from background), normalized against the average intensity at t = 0 with the overall average background intensity as the basal level (intensity = 0). Origami samples had been purified of extra staple strands by gel before the tests. See Supplementary Note 2 for details.

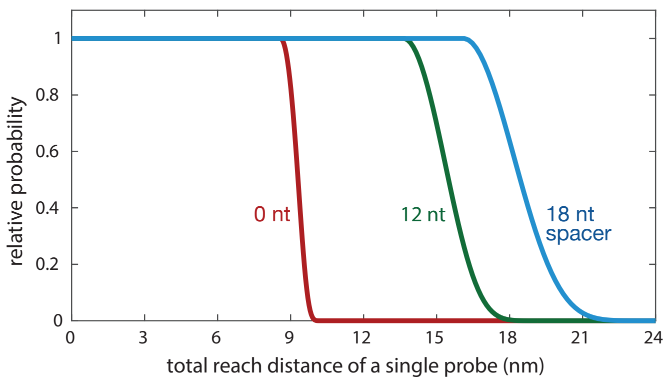

Supplementary Figure 8: **The Worm-Like Chain (WLC) model of DNA end-end length also fits probe reach data**. Estimation of the reach distance of a probe, oriented and with components as in Main Text Figure 4b. A plot of the probability densities of the reach distances of a probe for different lengths of spacers, calculated based on the end-to-end distances of the single and double-stranded portions using the worm-like-chain model. See Supplementary Note 3 for details.

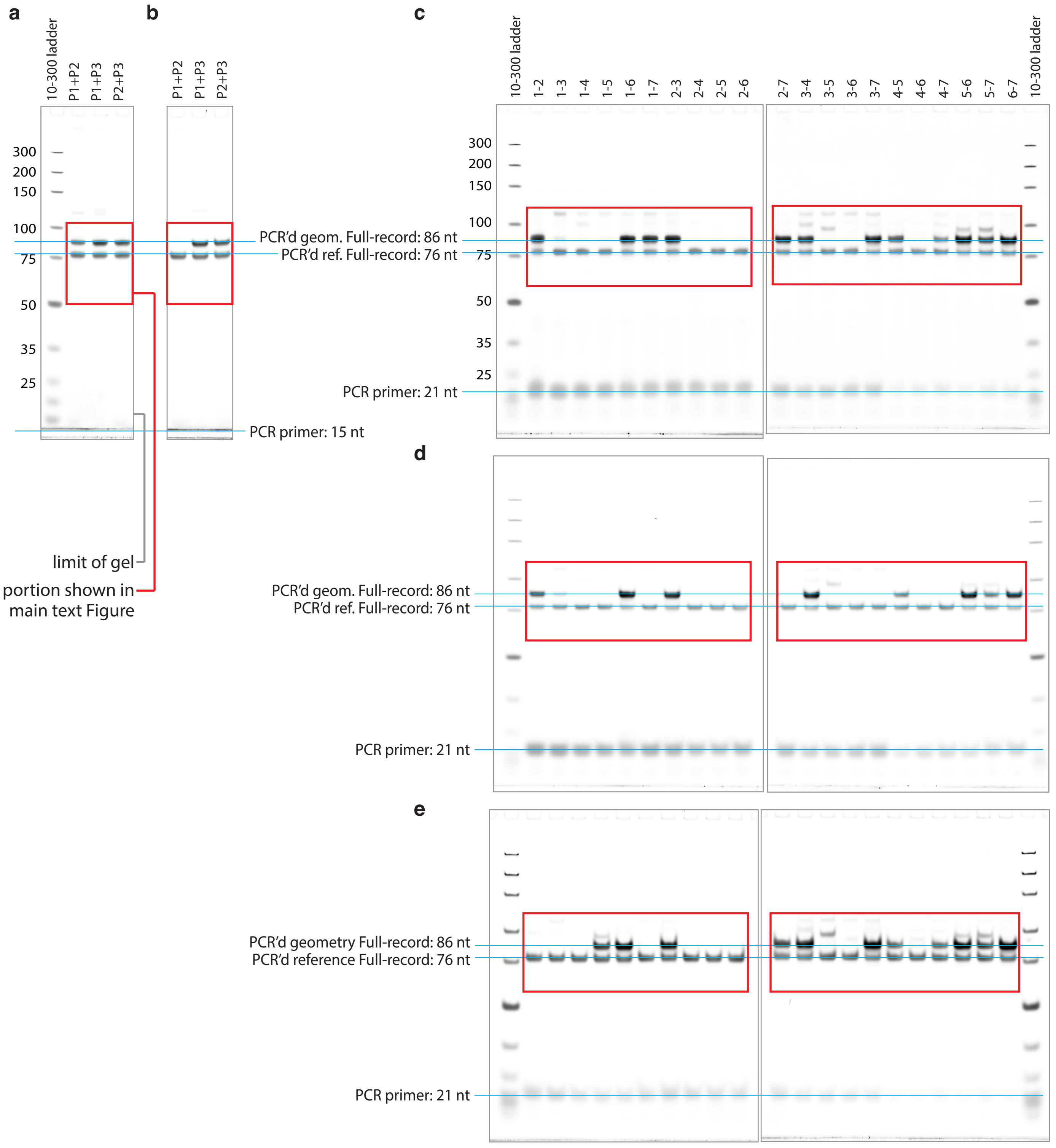

Supplementary Figure 9: **Full, un-cropped gel lanes for Main Text Figure 5**. Gels are labeled (a-e) in the same sequence as those of the main text Figure. Only the Cy5 channel (i.e., primer and extensions) is shown. Ladder, lanes, and main text figure cropping as noted.

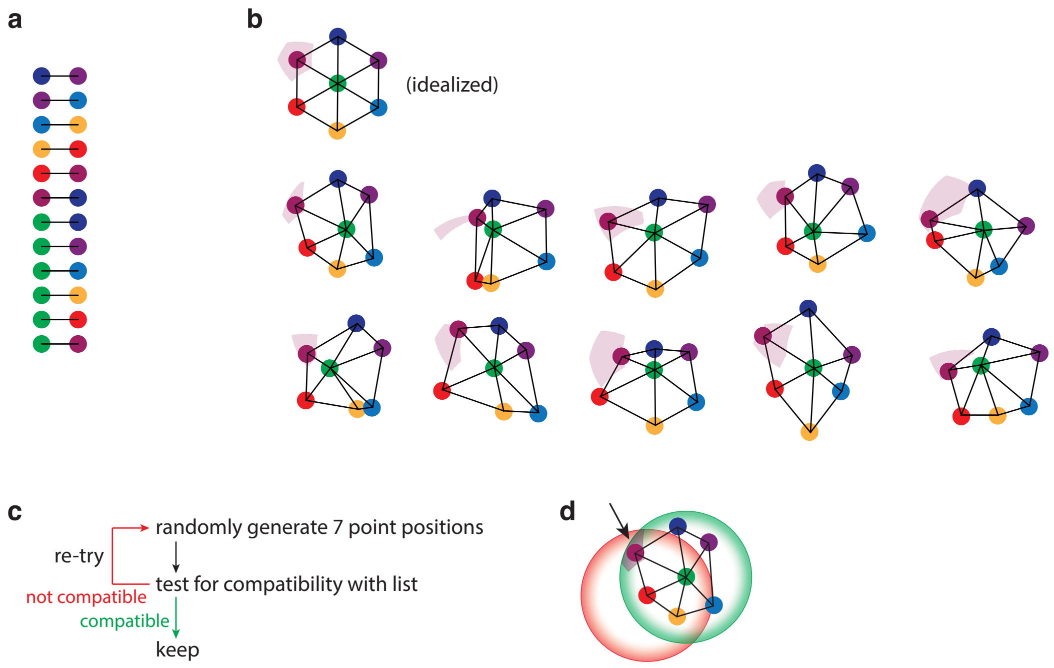

Supplementary Figure 10: **Reconstruction precision**. The code in Supplementary Note 4 draws an idealized graph from a list of connectivities (Main Text Figure 5 reconstructions), but a range of similar geometries may have been used to create the same Full-records and proximity list. We refer to the extent of this range of geometries as the precision of the method. (**a**) APR applied to a given geometry and probe set may generate the diagrammed set of proximities among 7 uniquely colored probes. These are in fact the same 12 of 21 possible proximities as those of the ideal hexagon in Main Text Figure 5c. (**b**) The code of Supplementary Note 4 generates the idealized geometry indicated. It is certainly compatible with the data, but so are the 10 other randomly-generated graphs shown. The relative positions of the probes are all similar, but their precise positions have some variation. (Graphs are rotated and reflected as necessary for easy comparison.) To further demonstrate precision in this example, a single probe in each case is depicted within a shaded range of positions. The shading describes the positions, given the positions of the 6 other probes as shown in each example, with which the list of connectivities is still compatible. (**c**) The graphs in (b) were randomly generated by applying the following simple algorithm, and represent 10 consecutively-generated compatible graphs. The test for compatibility is simply checking if the 21 possible proximities match the proximity list, given a cutoff radius (reach). (**d**) The range of positions for a single probe was calculated geometrically by drawing circles of a given reach around each probe and selecting the area that is within the circle for nearby probes and outside of the circle for farther points. Of the 6 such circles used in calculating the plausible range of the magenta probe (arrow), only 2 are shown for clarity: the magenta probe must be within reach of both green and red probes.

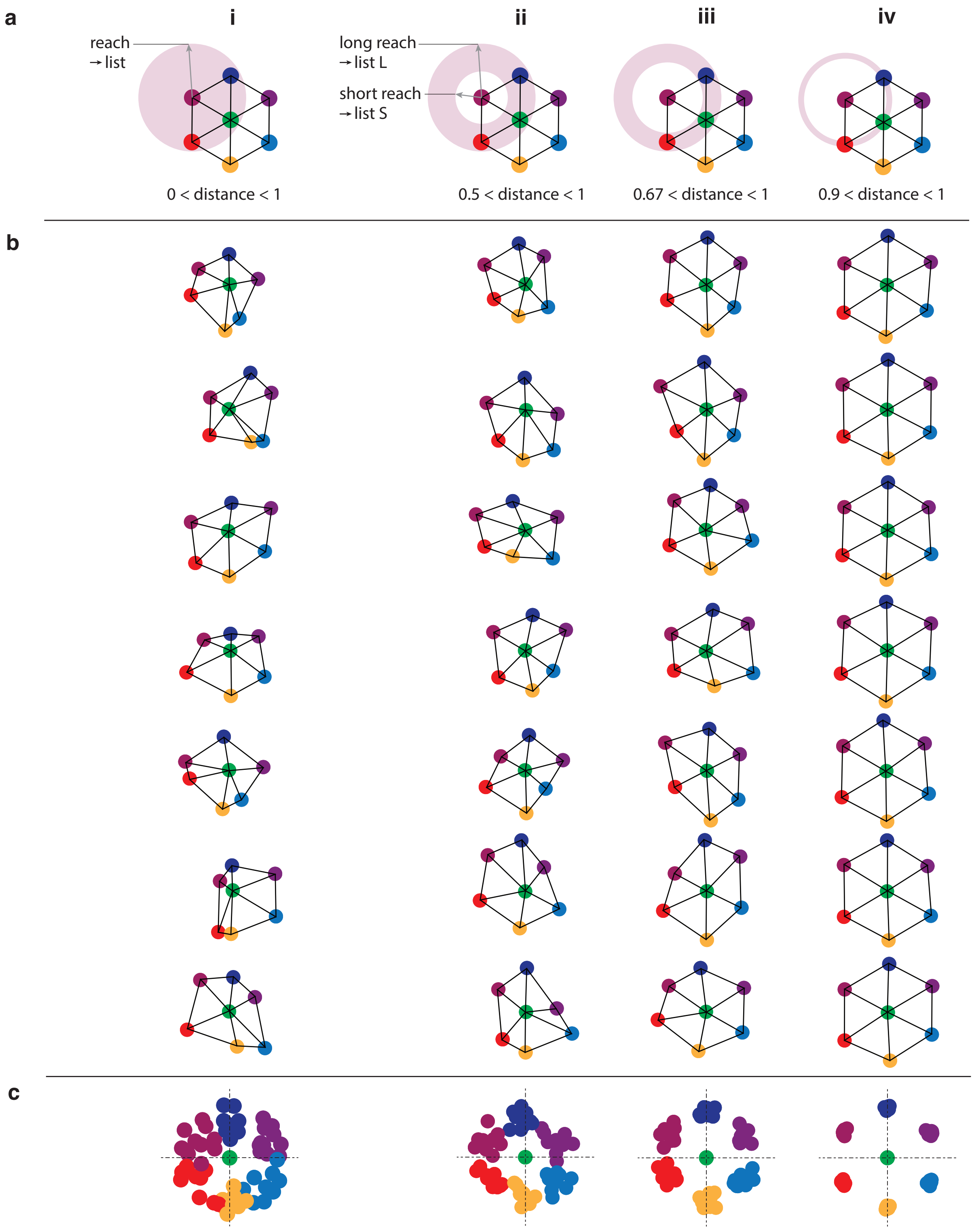

Supplementary Figure 11: **Reconstruction precision is improved by measurement with two or more probes of different reach**. (**a**) Full records generated between a given probe and any other (i) characterizes the separation as either within reach or not. During geometry reconstruction, this is interpreted as a maximum radius with which to discriminate pairs as nearby or not. Data from two separate measurements utilizing probes with different reach (ii-iv) allows one to specify each distance as very near, intermediate (within the shaded ring), or farther. Adjusting the relative reach of the two probe types changes the radius of each cutoff circle. (**b**) As in Supplementary Figure 10b, graphs consistent with the data can be randomly generated to give a impression of the precision, or reliability, of the reconstruction. Given a single probe measurement and resulting list (the same as that of Supplementary Figure 10a) yields the random graphs shown. If two probe measurements are made such that, in this case, the connections fall in between the reach of two probe types, the list of Supplementary Figure 10a represents the longer probe reach “list L,” and the list for the shorter probe reach (“List S”) would be empty. Reconstruction can take this into account in not allowing graphs where pairs from the longer list are too close. In the case of a short:long probe reach ratio of 0.5:1 (ii), this improves precision somewhat. Precision is further improved by utilizing more probe measurements or, as shown, by tightening the proximity range in which the connections appear (iii, iv). (**c**) The overlap of reconstruction points of 10 consecutive random reconstructions, including those shown in (b). Non-idealized geometries can be similarly refined.

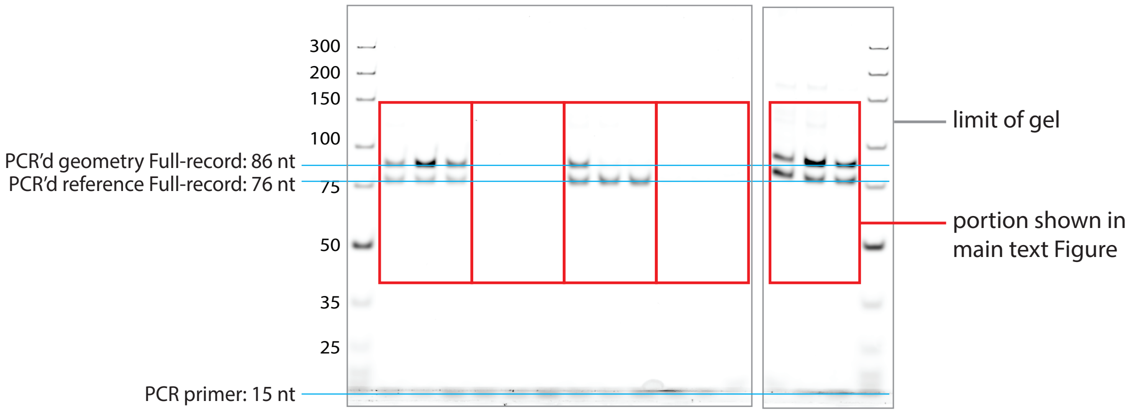

Supplementary Figure 12: **Full, un-cropped gel lanes for Main Text Figure 6b**. Only the Cy5 channel (i.e., the primer and extensions) is shown. Ladder, lanes, and main text figure cropping as noted.

Regular staples

Name	Start coords Helix#[base#]	End coords Helix#[base#]	Sequence
Staple-1[32]-3[31]	1[32]	3[31]	AGGCTCCAGAGGCTTTGAGGACACGGGTAA
Staple-3[32]-5[31]	3[32]	5[31]	AATACGTTTGAAAGAGGACAGACTGACCTT
Staple-5[32]-7[31]	5[32]	7[31]	CATCAAGTAAAACGAACTAACGAGTTGAGA
Staple-7[32]-9[31]	7[32]	9[31]	TTTAGGACAAATGCTTTAAACAATCAGGTC
Staple-9[32]-11[31]	9[32]	11[31]	TTTACCCCAACATGTTTTAAATTTCCATAT
Staple-11[32]-13[31]	11[32]	13[31]	AACAGTTTTGTACCAAAAACATTTTATTTC
Staple-13[32]-15[31]	13[32]	15[31]	AACGCAAAATCGATGAACGGTACCGGTTGA
Staple-15[32]-17[31]	15[32]	17[31]	TAATCAGCGGATTGACCGTAATCGTAACCG
Staple-17[32]-19[31]	17[32]	19[31]	TGCATCTTTCCCAGTCACGACGGCCTGCAG
Staple-19[32]-21[31]	19[32]	21[31]	GTCGACTTCGGCCAACGCGCGGGGTTTTTC
Staple-21[32]-23[31]	21[32]	23[31]	TTTTCACTCAAAGGGCGAAAAACCATCACC
Staple-23[32]-22[48]	23[32]	22[48]	CAAATCAAGTTTTTTGGGGTCGAAACGTGGA
Staple-0[47]-1[31]	0[47]	1[31]	AGAAAGGAACAACTAAAGGAATTCAAAAAAA
Staple-2[47]-0[48]	2[47]	0[48]	ACGGCTACAAAAGGAGCCTTTAATGTGAGAAT
Staple-4[47]-2[48]	4[47]	2[48]	GACCAACTAATGCCACTACGAAGGGGGTAGCA
Staple-6[47]-4[48]	6[47]	4[48]	TACGTTAAAGTAATCTTGACAAGAACCGAACT
Staple-8[47]-6[48]	8[47]	6[48]	ATCCCCCTATACCACATTCAACTAGAAAAATC
Staple-10[47]-8[48]	10[47]	8[48]	CTGTAGCTTGACTATTATAGTCAGTTCATTGA
Staple-12[47]-10[48]	12[47]	10[48]	TAAATCGGGATTCCCAATTCTGCGATATAATG
Staple-14[47]-12[48]	14[47]	12[48]	AACAAGAGGGATAAAAATTTTTAGCATAAAGC
Staple-16[47]-14[48]	16[47]	14[48]	ACAAACGGAAAAGCCCCAAAAACACTGGAGCA
Staple-18[47]-16[48]	18[47]	16[48]	CCAGGGTTGCCAGTTTGAGGGGACCCGTGGGA
Staple-20[47]-18[48]	20[47]	18[48]	TTAATGAACTAGAGGATCCCCGGGGGGTAACG
Staple-22[47]-20[48]	22[47]	20[48]	CTCCAACGCAGTGAGACGGGCAACCAGCTGCA
Staple-1[64]-3[63]	1[64]	3[63]	TTTATCAGGACAGCATCGGAACGACACCAACC
Staple-3[64]-5[63]	3[64]	5[63]	TAAAACGAGGTCAATCATAAGGGAACCGGATA
Staple-5[64]-7[63]	5[64]	7[63]	TTCATTACGTCAGGACGTTGGGAAATGCAGAT
Staple-7[64]-9[63]	7[64]	9[63]	ACATAACGGGAATCGTCATAAATAAAGCAAAG
Staple-9[64]-11[63]	9[64]	11[63]	CGGATTGCAGAGCTTAATTGCTGAAACGAGTA
Staple-11[64]-13[63]	11[64]	13[63]	GATTTAGTCAATAAAGCCTCAGAGAACCCTCA
Staple-13[64]-15[63]	13[64]	15[63]	TATATTTTGTCATTGCCTGAGAGTGGAAGATT
Staple-15[64]-17[63]	15[64]	17[63]	GTATAAGCCAACCCGTCGGATTCTGACGACAG
Staple-17[64]-19[63]	17[64]	19[63]	TATCGGCCGCAAGGCGATTAAGTTTACCGAGC
Staple-19[64]-21[63]	19[64]	21[63]	TCGAATTCGGGAAACCTGTCGTGCAGCTGATT
Staple-21[64]-23[63]	21[64]	23[63]	GCCCTTCAGAGTCCACTATTAAAGGGTGCCGT
Staple-23[64]-22[80]	23[64]	22[80]	AAAGCACTAAATCGGAACCCTAATCCAGTT
Staple-0[79]-1[63]	0[79]	1[63]	ACAACTTTCAACAGTTTCAGCGGATGTATCGG
Staple-2[79]-0[80]	2[79]	0[80]	CAGCGAAACTTGCTTTCGAGGTGTTGCTAA
Staple-4[79]-2[80]	4[79]	2[80]	GCGCAGACAAGAGGCAAAAGAATCCCTCAG
Staple-6[79]-4[80]	6[79]	4[80]	TTATACCACCAAATCAACGTAACGAACGAG
Staple-8[79]-6[80]	8[79]	6[80]	AATACTGCCCAAAAGGAATTACGTGGCTCA
Staple-10[79]-8[80]	10[79]	8[80]	GATGGCTTATCAAAAAGATTAAGAGCGTCC
Staple-12[79]-10[80]	12[79]	10[80]	AAATTAAGTTGACCATTAGATACTTTTGCG
Staple-14[79]-12[80]	14[79]	12[80]	GCTATCAGAAATGCAATGCCTGAATTAGCA
Staple-16[79]-14[80]	16[79]	14[80]	GCGAGTAAAAATATTTAAATTGTTACAAAG
Staple-18[79]-16[80]	18[79]	16[80]	GATGTGCTTCAGGAAGATCGCACAATGTGA
Staple-20[79]-18[80]	20[79]	18[80]	TTCCAGTCGTAATCATGGTCATAAAAGGGG
Staple-22[79]-20[80]	22[79]	20[80]	TGGAACAACCGCCTGGCCCTGAGGCCCGCT
Staple-1[96]-3[95]	1[96]	3[95]	AAACAGCTTTTTGCGGGATCGTCAACACTAAA
Staple-3[96]-5[95]	3[96]	5[95]	ACACTCATCCATGTTACTTAGCCGAAAGCTGC
Staple-5[96]-7[95]	5[96]	7[95]	TCATTCAGATGCGATTTTAAGAACAGGCATAG
Staple-7[96]-9[95]	7[96]	9[95]	TAAGAGCAAATGTTTAGACTGGATAGGAAGCC
Staple-9[96]-11[95]	9[96]	11[95]	CGAAAGACTTTGATAAGAGGTCATATTTCGCA
Staple-11[96]-13[95]	11[96]	13[95]	AATGGTCAACAGGCAAGGCAAAGAGTAATGTG
Staple-13[96]-15[95]	13[96]	15[95]	TAGGTAAACTATTTTTGAGAGATCAAACGTTA
Staple-15[96]-17[95]	15[96]	17[95]	ATATTTTGGCTTTCATCAACATTATCCAGCCA
Staple-17[96]-19[95]	17[96]	19[95]	GCTTTCCGATTACGCCAGCTGGCGGCTGTTTC
Staple-19[96]-21[95]	19[96]	21[95]	CTGTGTGATTGCGTTGCGCTCACTAGAGTTGC
Staple-21[96]-23[95]	21[96]	23[95]	AGCAAGCGTAGGGTTGAGTGTTGTAGGGAGCC
Staple-23[96]-22[112]	23[96]	22[112]	CCCGATTTAGAGCTTGACGGGGAAAAAGAATA
Staple-0[111]-1[95]	0[111]	1[95]	TAAATGAATTTTCTGTATGGGATTAATTTCTT
Staple-2[111]-0[112]	2[111]	0[112]	AAGGCCGCTGATACCGATAGTTGCGACGTTAG
Staple-4[111]-2[112]	4[111]	2[112]	GACCTGCTCTTTGACCCCCAGCGAGGGAGTTA
Staple-6[111]-4[112]	6[111]	4[112]	ATTACCTTTGAATAAGGCTTGCCCAAATCCGC
Staple-8[111]-6[112]	8[111]	6[112]	AATAGTAAACACTATCATAACCCTCATTGTGA
Staple-10[111]-8[112]	10[111]	8[112]	TTGCTCCTTTCAAATATCGCGTTTGAGGGGGT
Staple-12[111]-10[112]	12[111]	10[112]	TAAATCATATAACCTGTTTAGCTAACCTTTAA
Staple-14[111]-12[112]	14[111]	12[112]	GAGGGTAGGATTCAAAAGGGTGAGACATCCAA
Staple-16[111]-14[112]	16[111]	14[112]	TGTAGCCATTAAAATTCGCATTAAATGCCGGA
Staple-18[111]-16[112]	18[111]	16[112]	TCTTCGCTGCACCGCTTCTGGTGCGGCCTTCC
Staple-20[111]-18[112]	20[111]	18[112]	CACATTAAAATTGTTATCCGCTCATGCGGGCC
Staple-22[111]-20[112]	22[111]	20[112]	GCCCGAGAGTCCACGCTGGTTTGCAGCTAACT
Staple-1[128]-3[127]	1[128]	3[127]	TGACAACTCGCTGAGGCTTGCATTATACCA
Staple-3[128]-5[127]	3[128]	5[127]	AGCGCGATGATAAATTGTGTCGTGACGAGA
Staple-5[128]-7[127]	5[128]	7[127]	AACACCAAATTTCAACTTTAATCGTTTACC
Staple-7[128]-9[127]	7[128]	9[127]	AGACGACAAAGAAGTTTTGCCATAATTCGA
Staple-9[128]-11[127]	9[128]	11[127]	GCTTCAATCAGGATTAGAGAGTTATTTTCA
Staple-11[128]-13[127]	11[128]	13[127]	TTTGGGGATAGTAGTAGCATTAAAAGGCCG
Staple-13[128]-15[127]	13[128]	15[127]	GAGACAGCTAGCTGATAAATTAATTTTTGT
Staple-15[128]-17[127]	15[128]	17[127]	TAAATCAAAATAATTCGCGTCTCGGAAACC

Staple-17[128]-19[127]	17[128]	19[127]	AGGCAAAGGGAAGGGCGATCGGCAATTCCA
Staple-19[128]-21[127]	19[128]	21[127]	CACAACAGGTGCCTAATGAGTGCCCAGCAG
Staple-21[128]-23[127]	21[128]	23[127]	GCGAAAAATCCCTTATAAATCAAGCCGGCG
Staple-23[128]-23[159]	23[128]	23[159]	AACGTGGCGAGAAAGGAAGGGAAACCAGTAA
Staple-0[143]-1[127]	0[143]	1[127]	TCTAAAGTTTTGTCGTCTTTCCAGCCGACAA
Staple-2[143]-1[159]	2[143]	1[159]	ATATTCGGAACCATCGCCCACGCAGAGAAGGA
Staple-4[143]-3[159]	4[143]	3[159]	TCATCGCCAACAAAGTACAACGGACGCCAGCA
Staple-6[143]-5[159]	6[143]	5[159]	GATGGTTTGAACGAGTAGTAAATTTACCATTA
Staple-8[143]-7[159]	8[143]	7[159]	CTTTTGCAGATAAAAACCAAAATAAAGACTCC
Staple-10[143]-9[159]	10[143]	9[159]	CCAACAGGAGCGAACCAGACCGGAGCCTTTAC
Staple-12[143]-11[159]	12[143]	11[159]	TTCTACTACGCGAGCTGAAAAGGTTACCGCGC
Staple-14[143]-13[159]	14[143]	13[159]	CAACCGTTTCAAATCACCATCAATTCGAGCCA
Staple-16[143]-15[159]	16[143]	15[159]	GCCATCAAGCTCATTTTTTAACCACAAATCCA
Staple-18[143]-17[159]	18[143]	17[159]	CAACTGTTGCGCCATTCGCCATTCAAACATCA
Staple-20[143]-19[159]	20[143]	19[159]	AAGCCTGGTACGAGCCGGAAGCATAGATGATG
Staple-22[143]-21[159]	22[143]	21[159]	TCGGCAAATCCTGTTTGATGGTGGACCCTCAA
Staple-1[160]-2[144]	1[160]	2[144]	TTAGGATTGGCTGAGACTCCTCAATAACCGAT
Staple-3[160]-4[144]	3[160]	4[144]	TTGACAGGCCACCACCAGAGCCGCGATTTGTA
Staple-5[160]-6[144]	5[160]	6[144]	GCAAGGCCTCACCAGTAGCACCATGGGCTTGA
Staple-7[160]-8[144]	7[160]	8[144]	TTATTACGAAGAACTGGCATGATTGCGAGAGG
Staple-9[160]-10[144]	9[160]	10[144]	AGAGAGAAAAAAATGAAAATAGCAAGCAAACT
Staple-11[160]-12[144]	11[160]	12[144]	CCAATAGCTCATCGTAGGAATCATGGCATCAA
Staple-13[160]-14[144]	13[160]	14[144]	GTAATAAGTTAGGCAGAGGCATTTATGATATT
Staple-15[160]-16[144]	15[160]	16[144]	ATCGCAAGTATGTAAATGCTGATGATAGGAAC
Staple-17[160]-18[144]	17[160]	18[144]	AGAAAACAAAGAAGATGATGAAACAGGCTGCG
Staple-19[160]-20[144]	19[160]	20[144]	GCAATTCACATATTCCTGATTATCAAAGTGTA
Staple-21[160]-22[144]	21[160]	22[144]	TCAATATCGAACCTCAAATATCAATTCCGAAA
Staple-23[160]-22[176]	23[160]	22[176]	TAAAAGGGACATTCTGGCCAACAAAGCATC
Staple-0[175]-0[144]	0[175]	0[144]	TCCACAGACAGCCCTCATAGTTAGCGTAACGA
Staple-2[175]-0[176]	2[175]	0[176]	TATTAAGAAGCGGGGTTTTGCTCGTAGCAT
Staple-4[175]-2[176]	4[175]	2[176]	CACCAGAAAGGTTGAGGCAGGTCATGAAAG
Staple-6[175]-4[176]	6[175]	4[176]	CAGCAAAAGGAAACGTCACCAATGAGCCGC
Staple-8[175]-6[176]	8[175]	6[176]	ATACCCAACAGTATGTTAGCAAATTAGAGC
Staple-10[175]-8[176]	10[175]	8[176]	TTAACGTCTAACATAAAAACAGGTAACGGA
Staple-12[175]-10[176]	12[175]	10[176]	TTTTATTTAAGCAAATCAGATATTTTTTGT
Staple-14[175]-12[176]	14[175]	12[176]	CATGTAATAGAATATAAAGTACCAAGCCGT
Staple-16[175]-14[176]	16[175]	14[176]	TATAACTAACAAAGAACGCGAGAACGCCAA
Staple-18[175]-16[176]	18[175]	16[176]	CTGAGCAAAAATTAATTACATTTTGGGTTA
Staple-20[175]-18[176]	20[175]	18[176]	ATTATCATTCAATATAATCCTGACAATTAC
Staple-22[175]-20[176]	22[175]	20[176]	ACCTTGCTTGGTCAGTTGGCAAAGAGCGGA
Staple-1[192]-3[191]	1[192]	3[191]	GCGGATAACCTATTATTCTGAAACAGACGATT
Staple-3[192]-5[191]	3[192]	5[191]	GGCCTTGAAGAGCCACCACCCTCAGAAACCAT
Staple-5[192]-7[191]	5[192]	7[191]	CGATAGCATTGAGCCATTTGGGAACGTAGAAA
Staple-7[192]-9[191]	7[192]	9[191]	ATACATACCGAGGAAACGCAATAAGAAGCGCA
Staple-9[192]-11[191]	9[192]	11[191]	TTAGACGGCCAAATAAGAAACGATAGAAGGCT
Staple-11[192]-13[191]	11[192]	13[191]	TATCCGGTCTCATCGAGAACAAGCGACAAAAG
Staple-13[192]-15[191]	13[192]	15[191]	GTAAAGTAATCGCCATATTTAACAAAACTTTT
Staple-15[192]-17[191]	15[192]	17[191]	TCAAATATAACCTCCGGCTTAGGTAACAATTT
Staple-17[192]-19[191]	17[192]	19[191]	CATTTGAAGGCGAATTATTCATTTTTGTTTGG
Staple-19[192]-21[191]	19[192]	21[191]	ATTATACTAAGAAACCACCAGAAGTCAACAGT
Staple-21[192]-23[191]	21[192]	23[191]	TGAAAGGAGCAAATGAAAAATCTAGAGATAGA
Staple-23[192]-22[208]	23[192]	22[208]	ACCCTTCTGACCTGAAAGCGTAAGACGCTGAG
Staple-0[207]-1[191]	0[207]	1[191]	TCACCAGTACAAACTACAACGCCTAGTACCAG
Staple-2[207]-0[208]	2[207]	0[208]	TTTCGGAAGTGCCGTCGAGAGGGTGAGTTTCG
Staple-4[207]-2[208]	4[207]	2[208]	CCACCCTCTATTCACAAACAAATACCTGCCTA
Staple-6[207]-4[208]	6[207]	4[208]	TCACCGACGCACCGTAATCAGTAGCAGAACCG
Staple-8[207]-6[208]	8[207]	6[208]	AAGGAAACATAAAGGTGGCAACATTATCACCG
Staple-10[207]-8[208]	10[207]	8[208]	ATCCCAATGAGAATTAACTGAACAGTTACCAG
Staple-12[207]-10[208]	12[207]	10[208]	GTACCGCAATTCTAAGAACGCGAGTATTATTT
Staple-14[207]-12[208]	14[207]	12[208]	AATTGAGAATTCTGTCCAGACGACTAAACCAA
Staple-16[207]-14[208]	16[207]	14[208]	ACCTTTTTATTTTAGTTAATTTCATAGGGCTT
Staple-18[207]-16[208]	18[207]	16[208]	CGCGCAGATTACCTTTTTTAATGGGAGAGACT
Staple-20[207]-18[208]	20[207]	18[208]	GCGGAACATCTGAATAATGGAAGGTACAAAAT
Staple-22[207]-20[208]	22[207]	20[208]	AGCCAGCAATTGAGGAAGGTTATCATCATTTT
Staple-1[224]-3[223]	1[224]	3[223]	GTATAGCAAACAGTTAATGCCCAATCCTCA
Staple-3[224]-5[223]	3[224]	5[223]	TTAAAGCCAGAGCCGCCACCCTCGACAGAA
Staple-5[224]-7[223]	5[224]	7[223]	TCAAGTTTCATTAAAGGTGAATATAAAAGA
Staple-7[224]-9[223]	7[224]	9[223]	AACGCAAAGATAGCCGAACAAACCCTGAAC
Staple-9[224]-11[223]	9[224]	11[223]	AAAGTCACAAAATAAACAGCCAGCGTTTTA
Staple-11[224]-13[223]	11[224]	13[223]	GCGAACCTCCAAGAACGGGTATGACAATAA
Staple-13[224]-15[223]	13[224]	15[223]	ACAACATGCCAACGCTCAACAGTCTTCTGA
Staple-15[224]-17[223]	15[224]	17[223]	CCTAAATCAAAATCATAGGTCTAAACAGTA
Staple-17[224]-19[223]	17[224]	19[223]	CATAAATCTTTGAATACCAAGTGTTAGAAC
Staple-19[224]-21[223]	19[224]	21[223]	CTACCATAGTTTGAGTAACATTTAAAATAT
Staple-21[224]-23[223]	21[224]	23[223]	CTTTAGGGCCTGCAACAGTGCCAATACGTG
Staple-23[224]-22[240]	23[224]	22[240]	GCACAGACAATATTTTTGAATGGGGTCAGTA
Staple-0[239]-1[223]	0[239]	1[223]	AGGAACCCATGTACCGTAACACTTGATATAA
Staple-2[239]-0[240]	2[239]	0[240]	GCCCGTATCCGGAATAGGTGTATCAGCCCAAT
Staple-4[239]-2[240]	4[239]	2[240]	GCCTCCCTCAGAATGGAAAGCGCAGTAACAGT
Staple-6[239]-4[240]	6[239]	4[240]	GAAATTATTGCCTTTAGCGTCAGACCGGAACC
Staple-8[239]-6[240]	8[239]	6[240]	AAGTAAGCAGACACCACGGAATAATATTGACG
Staple-10[239]-8[240]	10[239]	8[240]	GCCAGTTAGAGGGTAATTGAGCGCTTTAAGAA
Staple-12[239]-10[240]	12[239]	10[240]	CTTATCATTCCCGACTTGCGGGAGCCTAATTT
Staple-14[239]-12[240]	14[239]	12[240]	AGTATAAAGTTCAGCTAATGCAGATGTCTTTC

Staple-16[239]-14[240]	16[239]	14[240]	GAATTTATTTAATGGTTTGAAATATTCTTACC
Staple-18[239]-16[240]	18[239]	16[240]	CCTGATTGCAATATATGTGAGTGATCAATAGT
Staple-20[239]-18[240]	20[239]	18[240]	ATTTTAAAATCAAAATTATTTGCACGGATTCG
Staple-22[239]-20[240]	22[239]	20[240]	TTAACACCAGCACTAACAACTAATCGTTATTA
Staple-1[256]-3[255]	1[256]	3[255]	CAGGAGGTGGGGTCAGTGCCTTGAGTCTCTGA
Staple-3[256]-5[255]	3[256]	5[255]	ATTTACCGGGAACCAGAGCCACCACTGTAGCG
Staple-5[256]-7[255]	5[256]	7[255]	CGTTTTCAAGGGAGGGAAGGTAAAGTTTATTT
Staple-7[256]-9[255]	7[256]	9[255]	TGTCACAATCTTACCGAAGCCCTTTAATATCA
Staple-9[256]-11[255]	9[256]	11[255]	GAGAGATAGAGCGTCTTTCCAGAGGTTTTGAA
Staple-11[256]-13[255]	11[256]	13[255]	GCCTTAAACCAATCAATAATCGGCACGCGCCT
Staple-13[256]-15[255]	13[256]	15[255]	GTTTATCAATATGCGTTATACAAACCGACCGT
Staple-15[256]-17[255]	15[256]	17[255]	GTGATAAAAAGACGCTGAGAAGAGATAACCTT
Staple-17[256]-19[255]	17[256]	19[255]	GCTTCTGTTCGGGAGAAACAATAACGTAAAAC
Staple-19[256]-21[255]	19[256]	21[255]	AGAAATAAAAATCCTTTGCCCGAAAGATTAGA
Staple-21[256]-23[255]	21[256]	23[255]	GCCGTCAAAAAACAGAGGTGAGGCCTATTAGT
Staple-23[256]-22[272]	23[256]	22[272]	CTTTAATGCGCGAACTGATAGCCCCACCAG
Staple-0[271]-1[255]	0[271]	1[255]	CCACCCTCATTTTCAGGGATAGCAACCGTACT
Staple-2[271]-0[272]	2[271]	0[272]	GTTTTAACTTAGTACCGCCACCCAGAGCCA
Staple-4[271]-2[272]	4[271]	2[272]	AAATCACCTTCCAGTAAGCGTCAGTAATAA
Staple-6[271]-4[272]	6[271]	4[272]	ACCGATTGTCGGCATTTTCGGTCATAATCA
Staple-8[271]-6[272]	8[271]	6[272]	AATAGCTATCAATAGAAAATTCAACATTCA
Staple-10[271]-8[272]	10[271]	8[272]	ACGCTAACACCCACAAGAATTGAAAATAGC
Staple-12[271]-10[272]	12[271]	10[272]	TGTAGAAATCAAGATTAGTTGCTCTTACCA
Staple-14[271]-12[272]	14[271]	12[272]	TTAGTATCACAATAGATAAGTCCACGAGCA
Staple-16[271]-14[272]	16[271]	14[272]	CTTAGATTTAAGGCGTTAAATAAAGCCTGT
Staple-18[271]-16[272]	18[271]	16[272]	CTTTTACAAAATCGTCGCTATTAGCGATAG
Staple-20[271]-18[272]	20[271]	18[272]	CTCGTATTAGAAATTGCGTAGATACAGTAC
Staple-22[271]-20[272]	22[271]	20[272]	CAGAAGATTAGATAATACATTTGTCGACAA

Staples for distance characterization, with 0 nt-spacer probe extensions

Name	Start coords Helix#[base#]	End coords Helix#[base#]	Sequence
Rec-0-P1.5'end	18[79]	16[80]	CTCAC AGGCCT T CGCTGG TTT GATGTGCTTCAGGAAGATCGCACAATGTGA
Rec-0-P2.5'end-6nm	17[96]	19[95]	CTCAC AGGCCT T CGCTGG TTT GCTTTCCGATTACGCCAGCTGGCGGCTGTTTC
Rec-0-P2.5'end-12nm	16[111]	14[112]	CTCAC AGGCCT T CGCTGG TTT TGTAGCCATTAAAATTCGCATTAAATGCCGGA
Rec-0-P2.5'end-18nm	15[128]	17[127]	CTCAC AGGCCT T CGCTGG TTT TAAATCAAAATAATTCGCGTCTCGGAAACC
Rec-0-P2.5'end-24nm	14[143]	13[159]	CTCAC AGGCCT T CGCTGG TTT CAACCGTTTCAAATCACCATCAATTCGAGCCA
Rec-0-P2.5'end-30nm	13[160]	14[144]	CTCAC AGGCCT T CGCTGG TTT GTAATAAGTTAGGCAGAGGCATTTATGATATT
Rec-0-P2.5'end-36nm	12[175]	10[176]	CTCAC AGGCCT T CGCTGG TTT TTTTATTTAAGCAAATCAGATATTTTTTGT
Rec-0-P1.3'end	20[79]	18[80]	TTCCAGTCGTAATCATGGTCATAAAAGGGG TTT CCAGCG /iSp9/ AGGCCT GTGAG AGATTGCTAGGTAGGT TTT
Rec-0-P2.3'end-6nm	15[96]	17[95]	ATATTTTGGCTTTCATCAACATTATCCAGCCA TTT CCAGCG /iSp9/ AGGCCT GTGAG AGAAAGCTGAGAGATG TTT
Rec-0-P2.3'end-12nm	18[111]	16[112]	TCTTCGCTGCACCGCTTCTGGTGCGGCCTTCC TTT CCAGCG /iSp9/ AGGCCT GTGAG AGAAAGCTGAGAGATG TTT
Rec-0-P2.3'end-18nm	13[128]	15[127]	GAGACAGCTAGCTGATAAATTAATTTTTGT TTT CCAGCG /iSp9/ AGGCCT GTGAG AGAAAGCTGAGAGATG TTT
Rec-0-P2.3'end-24nm	13[160]	14[144]	GTAATAAGTTAGGCAGAGGCATTTATGATATT TTT CCAGCG /iSp9/ AGGCCT GTGAG AGAAAGCTGAGAGATG TTT
Rec-0-P2.3'end-30nm	14[143]	13[159]	CAACCGTTTCAAATCACCATCAATTCGAGCCA TTT CCAGCG /iSp9/ AGGCCT GTGAG AGAAAGCTGAGAGATG TTT
Rec-0-P2.3'end-36nm	14[175]	12[176]	CATGTAATAGAATATAAAGTACCAAGCCGT TTT CCAGCG /iSp9/ AGGCCT GTGAG AGAAAGCTGAGAGATG TTT

Staples for distance characterization, with 12 nt-spacer probe extensions

Name	Start coords Helix#[base#]	End coords Helix#[base#]	Sequence
Rec-12-P1.5'end	18[79]	16[80]	CTCAC TCTTCTCACTAT AGGCCT T CGCTGG TTT GATGTGCTTCAGGAAGATCGCACAATGTGA
Rec-12-P2.5'end-6nm	17[96]	19[95]	CTCAC AACATATACATT AGGCCT T CGCTGG TTT GCTTTCCGATTACGCCAGCTGGCGGCTGTTTC
Rec-12-P2.5'end-12nm	16[111]	14[112]	CTCAC AACATATACATT AGGCCT T CGCTGG TTT TGTAGCCATTAAAATTCGCATTAAATGCCGGA
Rec-12-P2.5'end-18nm	15[128]	17[127]	CTCAC AACATATACATT AGGCCT T CGCTGG TTT TAAATCAAAATAATTCGCGTCTCGGAAACC
Rec-12-P2.5'end-24nm	14[143]	13[159]	CTCAC AACATATACATT AGGCCT T CGCTGG TTT CAACCGTTTCAAATCACCATCAATTCGAGCCA
Rec-12-P2.5'end-30nm	13[160]	14[144]	CTCAC AACATATACATT AGGCCT T CGCTGG TTT GTAATAAGTTAGGCAGAGGCATTTATGATATT
Rec-12-P2.5'end-36nm	12[175]	10[176]	CTCAC AACATATACATT AGGCCT T CGCTGG TTT TTTTATTTAAGCAAATCAGATATTTTTTGT
Rec-12-P2.5'end-42nm	11[192]	13[191]	CTCAC AACATATACATT AGGCCT T CGCTGG TTT TATCCGGTCTCATCGAGAACAAGCGACAAAAG
Rec-12-P2.5'end-48nm	10[207]	8[208]	CTCAC AACATATACATT AGGCCT T CGCTGG TTT ATCCCAATGAGAATTAACTGAACAGTTACCAG
Rec-12-P1.3'end	20[79]	18[80]	TTCCAGTCGTAATCATGGTCATAAAAGGGG TTT CCAGCG /iSp9/ AGGCCT ATAGTGAGAAGA GTGAG AGATTGCTAGGTAGGT TTT
Rec-12-P2.3'end-6nm	15[96]	17[95]	ATATTTTGGCTTTCATCAACATTATCCAGCCA TTT CCAGCG /iSp9/ AGGCCT AATGTATATGTT GTGAG AGAAAGCTGAGAGATG TTT
Rec-12-P2.3'end-12nm	18[111]	16[112]	TCTTCGCTGCACCGCTTCTGGTGCGGCCTTCC TTT CCAGCG /iSp9/ AGGCCT AATGTATATGTT GTGAG AGAAAGCTGAGAGATG TTT
Rec-12-P2.3'end-18nm	13[128]	15[127]	GAGACAGCTAGCTGATAAATTAATTTTTGT TTT CCAGCG /iSp9/ AGGCCT AATGTATATGTT GTGAG AGAAAGCTGAGAGATG TTT
Rec-12-P2.3'end-24nm	13[160]	14[144]	GTAATAAGTTAGGCAGAGGCATTTATGATATT TTT CCAGCG /iSp9/ AGGCCT AATGTATATGTT GTGAG AGAAAGCTGAGAGATG TTT
Rec-12-P2.3'end-30nm	14[143]	13[159]	CAACCGTTTCAAATCACCATCAATTCGAGCCA TTT CCAGCG /iSp9/ AGGCCT AATGTATATGTT GTGAG AGAAAGCTGAGAGATG TTT
Rec-12-P2.3'end-36nm	14[175]	12[176]	CATGTAATAGAATATAAAGTACCAAGCCGT TTT CCAGCG /iSp9/ AGGCCT AATGTATATGTT GTGAG AGAAAGCTGAGAGATG TTT
Rec-12-P2.3'end-42nm	9[192]	11[191]	TTAGACGGCCAAATAAGAAACGATAGAAGGCT TTT CCAGCG /iSp9/ AGGCCT AATGTATATGTT GTGAG AGAAAGCTGAGAGATG TTT
Rec-12-P2.3'end-48nm	12[207]	10[208]	GTACCGCAATTCTAAGAACGCGAGTATTATTT TTT CCAGCG /iSp9/ AGGCCT AATGTATATGTT GTGAG AGAAAGCTGAGAGATG TTT

Staples for distance characterization, for click chemistry linkage

Name	Start coords Helix#[base#]	End coords Helix#[base#]	Sequence
Rec-anchor-P1	18[79]	16[80]	CGTCTAGTCTGAGCATTG T GATGTGCTTCAGGAAGATCGCACAATGTGA
Rec-anchor-P2-6nm	17[96]	19[95]	CATACCTAGCTCATAAGC T GCTTTCCGATTACGCCAGCTGGCGGCTGTTTC
Rec-anchor-P2-12nm	16[111]	14[112]	CATACCTAGCTCATAAGC T TGTAGCCATTAAAATTCGCATTAAATGCCGGA
Rec-anchor-P2-18nm	15[128]	17[127]	CATACCTAGCTCATAAGC T TAAATCAAAATAATTCGCGTCTCGGAAACC
Rec-anchor-P2-24nm	14[143]	13[159]	CATACCTAGCTCATAAGC T CAACCGTTTCAAATCACCATCAATTCGAGCCA
Rec-anchor-P2-30nm	13[160]	14[144]	CATACCTAGCTCATAAGC T GTAATAAGTTAGGCAGAGGCATTTATGATATT
Rec-anchor-P2-36nm	12[175]	10[176]	CATACCTAGCTCATAAGC T TTTTATTTAAGCAAATCAGATATTTTTTGT
Rec-anchor-P2-42nm	11[192]	13[191]	CATACCTAGCTCATAAGC T TATCCGGTCTCATCGAGAACAAGCGACAAAAG
Rec-anchor-P2-48nm	10[207]	8[208]	CATACCTAGCTCATAAGC T ATCCCAATGAGAATTAACTGAACAGTTACCAG

Staples for triangle-vs-line tests

Name	Start coords Helix#[base#]	End coords Helix#[base#]	Sequence
Rec-anchor-P1	18[79]	16[80]	CGTCTAGTCTGAGCATTG T GATGTGCTTCAGGAAGATCGCACAATGTGA
Rec-anchor-P2-30nm	13[160]	14[144]	CATACCTAGCTCATAAGC T GTAATAAGTTAGGCAGAGGCATTTATGATATT
Rec-anchor-P3-30nm-tri	8[79]	6[80]	GCCACATCCTGGATCTCG T AATACTGCCCAAAAGGAATTACGTGGCTCA
Rec-anchor-P3-30nm-line	8[239]	6[240]	GCCACATCCTGGATCTCG T AAGTAAGCAGACACCACGGAATAATATTGACG
Rec-anchor-P1_on_S2	13[160]	14[144]	CGTCTAGTCTGAGCATTG T GTAATAAGTTAGGCAGAGGCATTTATGATATT
Rec-anchor-P3_on_S2	13[160]	14[144]	GCCACATCCTGGATCTCG T GTAATAAGTTAGGCAGAGGCATTTATGATATT
Rec-anchor-P2_on_S1	18[79]	16[80]	CATACCTAGCTCATAAGC T GATGTGCTTCAGGAAGATCGCACAATGTGA
Rec-anchor-P2_on_S3	8[239]	6[240]	CATACCTAGCTCATAAGC T AAGTAAGCAGACACCACGGAATAATATTGACG

Staples with probe extensions for reference probe sites

Name	Start coords Helix#[base#]	End coords Helix#[base#]	Sequence
Ref-13-P1.5'end	5[64]	7[63]	CTCAC CTCCTCATACATC TGCGCA T CGCTGG TTT TTCATTACGTCAGGACGTTGGGAAATGCAGAT
Ref-13-P2.5'end	4[79]	2[80]	CTCAC TTCTAATTCACAC TGCGCA T CGCTGG TTT GCGCAGACAAGAGGCAAAAGAATCCCTCAG
Ref-13-P1.3'end	3[64]	5[63]	TAAAACGAGGTCAATCATAAGGGAACCGGATA TTT CCAGCG /iSp9/ TGCGCA GATGTATGAGGAG GTGAG AAGATGTCGAGTATGG TTT
Ref-13-P2.3'end	6[79]	4[80]	TTATACCACCAAATCAACGTAACGAACGAG TTT CCAGCG /iSp9/ TGCGCA GTGTGAATTAGAA GTGAG AAGTATCGAAGGGAGT TTT

Staples for 7-point hexagonal geometries

Name	Start coords Helix#[base#]	End coords Helix#[base#]	Sequence
Hex-anch-24nm-P1	4[143]	3[159]	CGTCTAGTCTGAGCATTG T TCATCGCCAACAAAGTACAACGGACGCCAGCA
Hex-anch-24nm-P2	8[79]	6[80]	CATACCTAGCTCATAAGC T AATACTGCCCAAAAGGAATTACGTGGCTCA
Hex-anch-24nm-P3	16[79]	14[80]	GCCACATCCTGGATCTCG T GCGAGTAAAAATATTTAAATTGTTACAAAG
Hex-anch-24nm-P4	20[143]	19[159]	CGGGTGGACTGCCTACGG T AAGCCTGGTACGAGCCGGAAGCATAGATGATG
Hex-anch-24nm-P5	16[207]	14[208]	GCGCATGTGTACTAGCAC T ACCTTTTTATTTTAGTTAATTTCATAGGGCTT
Hex-anch-24nm-P6	8[207]	6[208]	GCCGGGAGAGTTTTTGGG T AAGGAAACATAAAGGTGGCAACATTATCACCG
Hex-anch-24nm-P7	12[143]	11[159]	GTTGATAAGTCAGTATGC T TTCTACTACGCGAGCTGAAAAGGTTACCGCGC
Hex-anch-24nm-varP1	12[271]	10[272]	CGTCTAGTCTGAGCATTG T TGTAGAAATCAAGATTAGTTGCTCTTACCA
Hex-anch-24nm-varP2	4[271]	2[272]	CATACCTAGCTCATAAGC T AAATCACCTTCCAGTAAGCGTCAGTAATAA
Hex-anch-24nm-varP7	12[271]	10[272]	GTTGATAAGTCAGTATGC T TGTAGAAATCAAGATTAGTTGCTCTTACCA

Cy3 & related strands for supplementary origami figure

Name	Start coords Helix#[base#]	End coords Helix#[base#]	Sequence
Cy3_anch-0[239]-1[223]	0[239]	1[223]	AGGAACCCATGTACCGTAACACTTGATATAA TAACATTCCTAACTTCTCATA
Cy3_anch-1[224]-3[223]	1[224]	3[223]	GTATAGCAAACAGTTAATGCCCAATCCTCA TAACATTCCTAACTTCTCATA
Cy3_anch-3[224]-5[223]	3[224]	5[223]	TTAAAGCCAGAGCCGCCACCCTCGACAGAA TAACATTCCTAACTTCTCATA
Cy3_anch-5[224]-7[223]	5[224]	7[223]	TCAAGTTTCATTAAAGGTGAATATAAAAGA TAACATTCCTAACTTCTCATA
Cy3_anch-7[224]-9[223]	7[224]	9[223]	AACGCAAAGATAGCCGAACAAACCCTGAAC TAACATTCCTAACTTCTCATA
Cy3_anch-9[224]-11[223]	9[224]	11[223]	AAAGTCACAAAATAAACAGCCAGCGTTTTA TAACATTCCTAACTTCTCATA
Cy3_anch-11[224]-13[223]	11[224]	13[223]	GCGAACCTCCAAGAACGGGTATGACAATAA TAACATTCCTAACTTCTCATA
Cy3_anch-13[224]-15[223]	13[224]	15[223]	ACAACATGCCAACGCTCAACAGTCTTCTGA TAACATTCCTAACTTCTCATA
Cy3_anch-15[224]-17[223]	15[224]	17[223]	CCTAAATCAAAATCATAGGTCTAAACAGTA TAACATTCCTAACTTCTCATA
Cy3_anch-17[224]-19[223]	17[224]	19[223]	CATAAATCTTTGAATACCAAGTGTTAGAAC TAACATTCCTAACTTCTCATA
Cy3_anch-19[224]-21[223]	19[224]	21[223]	CTACCATAGTTTGAGTAACATTTAAAATAT TAACATTCCTAACTTCTCATA
Cy3_anch-21[224]-23[223]	21[224]	23[223]	CTTTAGGGCCTGCAACAGTGCCAATACGTG TAACATTCCTAACTTCTCATA
Cy3_anch-2[239]-0[240]	2[239]	0[240]	GCCCGTATCCGGAATAGGTGTATCAGCCCAAT TAACATTCCTAACTTCTCATA
Cy3_anch-4[239]-2[240]	4[239]	2[240]	GCCTCCCTCAGAATGGAAAGCGCAGTAACAGT TAACATTCCTAACTTCTCATA
Cy3_anch-6[239]-4[240]	6[239]	4[240]	GAAATTATTGCCTTTAGCGTCAGACCGGAACC TAACATTCCTAACTTCTCATA
Cy3_anch-8[239]-6[240]	8[239]	6[240]	AAGTAAGCAGACACCACGGAATAATATTGACG TAACATTCCTAACTTCTCATA
Cy3_anch-10[239]-8[240]	10[239]	8[240]	GCCAGTTAGAGGGTAATTGAGCGCTTTAAGAA TAACATTCCTAACTTCTCATA
Cy3_anch-12[239]-10[240]	12[239]	10[240]	CTTATCATTCCCGACTTGCGGGAGCCTAATTT TAACATTCCTAACTTCTCATA
Cy3_anch-14[239]-12[240]	14[239]	12[240]	AGTATAAAGTTCAGCTAATGCAGATGTCTTTC TAACATTCCTAACTTCTCATA
Cy3_anch-16[239]-14[240]	16[239]	14[240]	GAATTTATTTAATGGTTTGAAATATTCTTACC TAACATTCCTAACTTCTCATA
Cy3_anch-18[239]-16[240]	18[239]	16[240]	CCTGATTGCAATATATGTGAGTGATCAATAGT TAACATTCCTAACTTCTCATA
Cy3_anch-20[239]-18[240]	20[239]	18[240]	ATTTTAAAATCAAAATTATTTGCACGGATTCG TAACATTCCTAACTTCTCATA
Cy3_anch-22[239]-20[240]	22[239]	20[240]	TTAACACCAGCACTAACAACTAATCGTTATTA TAACATTCCTAACTTCTCATA
Cy3_anch-23[224]-22[240]	23[224]	22[240]	GCACAGACAATATTTTTGAATGGGGTCAGTA TAACATTCCTAACTTCTCATA
Cy3_anch-0[271]-1[255]	0[271]	1[255]	CCACCCTCATTTTCAGGGATAGCAACCGTACT TAACATTCCTAACTTCTCATA
Cy3_anch-1[256]-3[255]	1[256]	3[255]	CAGGAGGTGGGGTCAGTGCCTTGAGTCTCTGA TAACATTCCTAACTTCTCATA
Cy3_anch-7[256]-9[255]	7[256]	9[255]	TGTCACAATCTTACCGAAGCCCTTTAATATCA TAACATTCCTAACTTCTCATA
Cy3_anch-9[256]-11[255]	9[256]	11[255]	GAGAGATAGAGCGTCTTTCCAGAGGTTTTGAA TAACATTCCTAACTTCTCATA
Cy3_anch-11[256]-13[255]	11[256]	13[255]	GCCTTAAACCAATCAATAATCGGCACGCGCCT TAACATTCCTAACTTCTCATA
Cy3_anch-13[256]-15[255]	13[256]	15[255]	GTTTATCAATATGCGTTATACAAACCGACCGT TAACATTCCTAACTTCTCATA
Cy3_anch-15[256]-17[255]	15[256]	17[255]	GTGATAAAAAGACGCTGAGAAGAGATAACCTT TAACATTCCTAACTTCTCATA
Cy3_anch-21[256]-23[255]	21[256]	23[255]	GCCGTCAAAAAACAGAGGTGAGGCCTATTAGT TAACATTCCTAACTTCTCATA
Cy3_anch-2[271]-0[272]	2[271]	0[272]	GTTTTAACTTAGTACCGCCACCCAGAGCCA TAACATTCCTAACTTCTCATA
Cy3_anch-4[271]-2[272]	4[271]	2[272]	AAATCACCTTCCAGTAAGCGTCAGTAATAA TAACATTCCTAACTTCTCATA
Cy3_anch-6[271]-4[272]	6[271]	4[272]	ACCGATTGTCGGCATTTTCGGTCATAATCA TAACATTCCTAACTTCTCATA
Cy3_anch-8[271]-6[272]	8[271]	6[272]	AATAGCTATCAATAGAAAATTCAACATTCA TAACATTCCTAACTTCTCATA
Cy3_anch-10[271]-8[272]	10[271]	8[272]	ACGCTAACACCCACAAGAATTGAAAATAGC TAACATTCCTAACTTCTCATA
Cy3_anch-12[271]-10[272]	12[271]	10[272]	TGTAGAAATCAAGATTAGTTGCTCTTACCA TAACATTCCTAACTTCTCATA
Cy3_anch-14[271]-12[272]	14[271]	12[272]	TTAGTATCACAATAGATAAGTCCACGAGCA TAACATTCCTAACTTCTCATA
Cy3_anch-16[271]-14[272]	16[271]	14[272]	CTTAGATTTAAGGCGTTAAATAAAGCCTGT TAACATTCCTAACTTCTCATA
Cy3_anch-18[271]-16[272]	18[271]	16[272]	CTTTTACAAAATCGTCGCTATTAGCGATAG TAACATTCCTAACTTCTCATA
Cy3_anch-20[271]-18[272]	20[271]	18[272]	CTCGTATTAGAAATTGCGTAGATACAGTAC TAACATTCCTAACTTCTCATA
Cy3_anch-22[271]-20[272]	22[271]	20[272]	CAGAAGATTAGATAATACATTTGTCGACAA TAACATTCCTAACTTCTCATA
Cy3_anch-23[256]-22[272]	23[256]	22[272]	CTTTAATGCGCGAACTGATAGCCCCACCAG TAACATTCCTAACTTCTCATA

Cy3_strand	TATGAGAAGTTAGGAATGTTA/3Cy3Sp/

Biotin & related staples for supplementary origami figure

Name	Start coords Helix#[base#]	End coords Helix#[base#]	Sequence
BT-4[63]-6[56]	4[63]	6[56]	/5Biosg/ATAAGGGAACCGGATATTCATTACGTCAGGACGTTGGGAA
BT-4[127]-6[120]	4[127]	6[120]	/5Biosg/TTGTGTCGTGACGAGAAACACCAAATTTCAACTTTAAT
BT-4[191]-6[184]	4[191]	6[184]	/5Biosg/CACCCTCAGAAACCATCGATAGCATTGAGCCATTTGGGAA
BT-4[255]-6[248]	4[255]	6[248]	/5Biosg/AGCCACCACTGTAGCGCGTTTTCAAGGGAGGGAAGGTAAA
BT-18[63]-20[56]	18[63]	20[56]	/5Biosg/ATTAAGTTTACCGAGCTCGAATTCGGGAAACCTGTCGTGC
BT-18[127]-20[120]	18[127]	20[120]	/5Biosg/GCGATCGGCAATTCCACACAACAGGTGCCTAATGAGTG
BT-18[191]-20[184]	18[191]	20[184]	/5Biosg/ATTCATTTTTGTTTGGATTATACTAAGAAACCACCAGAAG
BT-18[255]-20[248]	18[255]	20[248]	/5Biosg/AACAATAACGTAAAACAGAAATAAAAATCCTTTGCCCGAA
BT_helper-1[64]-4[64]	1[64]	4[64]	TTTATCAGGACAGCATCGGAACGACACCAACCTAAAACGAGGTCAATC
BT_helper-1[128]-4[128]	1[128]	4[128]	TGACAACTCGCTGAGGCTTGCATTATACCAAGCGCGATGATAAA
BT_helper-1[192]-4[192]	1[192]	4[192]	GCGGATAACCTATTATTCTGAAACAGACGATTGGCCTTGAAGAGCCAC
BT_helper-1[256]-4[256]	1[256]	4[256]	CAGGAGGTGGGGTCAGTGCCTTGAGTCTCTGAATTTACCGGGAACCAG
BT_helper-7[56]-9[63]	7[56]	9[63]	ATGCAGATACATAACGGGAATCGTCATAAATAAAGCAAAG
BT_helper-7[120]-9[127]	7[120]	9[127]	CGTTTACCAGACGACAAAGAAGTTTTGCCATAATTCGA
BT_helper-7[184]-9[191]	7[184]	9[191]	CGTAGAAAATACATACCGAGGAAACGCAATAAGAAGCGCA
BT_helper-7[248]-9[255]	7[248]	9[255]	GTTTATTTTGTCACAATCTTACCGAAGCCCTTTAATATCA
BT_helper-15[64]-18[64]	15[64]	18[64]	GTATAAGCCAACCCGTCGGATTCTGACGACAGTATCGGCCGCAAGGCG
BT_helper-15[128]-18[128]	15[128]	18[128]	TAAATCAAAATAATTCGCGTCTCGGAAACCAGGCAAAGGGAAGG
BT_helper-15[192]-18[192]	15[192]	18[192]	TCAAATATAACCTCCGGCTTAGGTAACAATTTCATTTGAAGGCGAATT
BT_helper-15[256]-18[256]	15[256]	18[256]	GTGATAAAAAGACGCTGAGAAGAGATAACCTTGCTTCTGTTCGGGAGA
BT_helper-21[56]-23[63]	21[56]	23[63]	AGCTGATTGCCCTTCAGAGTCCACTATTAAAGGGTGCCGT
BT_helper-21[120]-23[127]	21[120]	23[127]	CCCAGCAGGCGAAAAATCCCTTATAAATCAAGCCGGCG
BT_helper-21[184]-23[191]	21[184]	23[191]	TCAACAGTTGAAAGGAGCAAATGAAAAATCTAGAGATAGA
BT_helper-21[248]-23[255]	21[248]	23[255]	AGATTAGAGCCGTCAAAAAACAGAGGTGAGGCCTATTAGT

Edge staples for supplementary origami figure

Name	Start coords Helix#[base#]	End coords Helix#[base#]	Sequence
EdgR-0[295]-1[295]	0[295]	1[295]	CAGAACCGCCACCCTCTCAGAACCGCCACCCT
EdgR-2[295]-3[295]	2[295]	3[295]	ATACAGGAGTGTACTGTACATGGCTTTTGATG
EdgR-4[295]-5[295]	4[295]	5[295]	CGTTTGCCATCTTTTCATAGCCCCCTTATTAG
EdgR-6[295]-7[295]	6[295]	7[295]	CAAAGACAAAAGGGCGTATGGTTTACCAGCGC
EdgR-8[295]-9[295]	8[295]	9[295]	AGAGCAAGAAACAATGGTTAAGCCCAATAATA
EdgR-10[295]-11[295]	10[295]	11[295]	CAATTTTATCCTGAATATTTTGCACCCAGCTA
EdgR-12[295]-13[295]	12[295]	13[295]	TATCCCATCCTAATTTTGAACAAGAAAAATAA
EdgR-14[295]-15[295]	14[295]	15[295]	CATAATTACTAGAAAAGAATAAACACCGGAAT
EdgR-16[295]-17[295]	16[295]	17[295]	AATCCTTGAAAACATAATTAATTTTCCCTTAG
EdgR-18[295]-19[295]	18[295]	19[295]	AGATGAATATACAGTATTTCAGGTTTAACGTC
EdgR-20[295]-21[295]	20[295]	21[295]	AGACTTTACAAACAATAGGATTTAGAAGTATT
EdgR-22[295]-23[295]	22[295]	23[295]	AAAAATACCGAACGAACTAAAACATCGCCATT
EdgL-1[8]-0[8]	1[8]	0[8]	TCACGTTGAAAATCTCGCGAATAATAATTTTT
EdgL-3[8]-2[8]	3[8]	2[8]	AGGAAGTTTCCATTAATAAAGACTTTTTCATG
EdgL-5[8]-4[8]	5[8]	4[8]	CAGGCGCATAGGCTGGTGAACGGTGTACAGAC
EdgL-7[8]-6[8]	7[8]	6[8]	GGTAGAAAGATTCATCGAACAACATTATTACA
EdgL-9[8]-8[8]	9[8]	8[8]	TGACCATAAATCAAAAGTTCAGAAAACGAGAA
EdgL-11[8]-10[8]	11[8]	10[8]	GTGTCTGGAAGTTTCAATGCAACTAAAGTACG
EdgL-13[8]-12[8]	13[8]	12[8]	TTTTGCGGGAGAAGCCTATGACCCTGTAATAC
EdgL-15[8]-14[8]	15[8]	14[8]	GTCAATCATATGTACCATCGTAAAACTAGCAT
EdgL-17[8]-16[8]	17[8]	16[8]	GTGTAGATGGGCGCATGGGATAGGTCACGTTG
EdgL-19[8]-18[8]	19[8]	18[8]	AGTGCCAAGCTTGCATTTGTAAAACGACGGCC
EdgL-21[8]-20[8]	21[8]	20[8]	TATTGGGCGCCAGGGTGGAGAGGCGGTTTGCG
EdgL-23[8]-22[8]	23[8]	22[8]	TGGCCCACTACGTGAACCGTCTATCAGGGCGA

Supplementary Table 1: **Origami regular and modified staples**. Regular (i.e., for construction of the origami structure itself) and alternative staples (i.e., containing probe extensions or other modifications). Subsets of regular staples are replaced by probes or other special staples for different tests, per Supplementary Figures 5 and 6. Start and end coordinates denote the 5' and 3' ends, respectively, as appears in Supplementary Figure 5. Note that the biotin and related staples for Supplementary Figure 7 have modified routing paths, and hence do not have fully matching coordinates as regular staples; see Jungmann, et al.[2] for diagram.

Probes and linkers for click chemistry-coupled distance characterization

Name	Sequence
P1-azide	CTCAC TCTTCTCACTATCTCTCT AGGCCT T CGCTGG TT/iAzideN/TT CCAGCG /iSp9/ AGGCCT AGAGAGATAGTGAGAAGA GTGAG AGATTGCTAGGTAGGT TTT
P2-azide	CTCAC CACATCCCTATCTACATT AGGCCT T CGCTGG TT/iAzideN/TT CCAGCG /iSp9/ AGGCCT AATGTAGATAGGGATGTG GTGAG AGAAAGCTGAGAGATG TTT
P1-linker	(alkyne) TTT CAATGCTCAGACTAGACG
P2-linker	(alkyne) TTT GCTTATGAGCTAGGTATG

Probes and linkers for click chemistry-coupled 30 nm triangle-vs-line geometry

Name	Sequence
P1-azide	CTCAC TCTTCTCACTATCTCTCT AGGCCT T CGCTGG TT/iAzideN/TT CCAGCG /iSp9/ AGGCCT AGAGAGATAGTGAGAAGA GTGAG AGATTGCTAGGTAGGT TTT
P2-azide	CTCAC CACATCCCTATCTACATT AGGCCT T CGCTGG TT/iAzideN/TT CCAGCG /iSp9/ AGGCCT AATGTAGATAGGGATGTG GTGAG AGAAAGCTGAGAGATG TTT
P3-azide	CTCAC ACACTCTTCAACATATAT AGGCCT T CGCTGG TT/iAzideN/TT CCAGCG /iSp9/ AGGCCT ATATATGTTGAAGAGTGT GTGAG AGTGGATGATCAAGAG TTT
P1-linker	(alkyne) TTT CAATGCTCAGACTAGACG
P2-linker	(alkyne) TTT GCTTATGAGCTAGGTATG
P3-linker	(alkyne) TTT CGAGATCCAGGATGTGGC

Probes and linkers for click chemistry-coupled 24 nm hexagonal grid

Name	Sequence
Hex-P1-azide	CTCAC AACATATACATT AGGCCT T CGCTGG TT/iAzideN/TT CCAGCG /iSp9/ AGGCCT AATGTATATGTT GTGAG AGATTGCTAGGTAGGT TTT
Hex-P2-azide	CTCAC AACATATACATT AGGCCT T CGCTGG TT/iAzideN/TT CCAGCG /iSp9/ AGGCCT AATGTATATGTT GTGAG AGAAAGCTGAGAGATG TTT
Hex-P3-azide	CTCAC AACATATACATT AGGCCT T CGCTGG TT/iAzideN/TT CCAGCG /iSp9/ AGGCCT AATGTATATGTT GTGAG AGTGGATGATCAAGAG TTT
Hex-P4-azide	CTCAC AACATATACATT AGGCCT T CGCTGG TT/iAzideN/TT CCAGCG /iSp9/ AGGCCT AATGTATATGTT GTGAG ATGTGTAAGGATGAAG TTT
Hex-P5-azide	CTCAC AACATATACATT AGGCCT T CGCTGG TT/iAzideN/TT CCAGCG /iSp9/ AGGCCT AATGTATATGTT GTGAG ATGAAGAAGGGGTTTG TTT
Hex-P6-azide	CTCAC AACATATACATT AGGCCT T CGCTGG TT/iAzideN/TT CCAGCG /iSp9/ AGGCCT AATGTATATGTT GTGAG AGTAATAGTGGTGGAG TTT
Hex-P7-azide	CTCAC AACATATACATT AGGCCT T CGCTGG TT/iAzideN/TT CCAGCG /iSp9/ AGGCCT AATGTATATGTT GTGAG ATGTCTATTGTGAGGG TTT
Hex-P1-linker	(alkyne) TTT CAATGCTCAGACTAGACG
Hex-P2-linker	(alkyne) TTT GCTTATGAGCTAGGTATG
Hex-P3-linker	(alkyne) TTT CGAGATCCAGGATGTGGC
Hex-P4-linker	(alkyne) TTT CCGTAGGCAGTCCACCCG
Hex-P5-linker	(alkyne) TTT GTGCTAGTACACATGCGC
Hex-P6-linker	(alkyne) TTT CCCAAAAACTCTCCCGGC
Hex-P7-linker	(alkyne) TTT GCATACTGACTTATCAAC

De/re-activator and recording and PCR primers for state change test on origami

Name	Sequence
Deactivator for P3	AAA CTC TTGATCATCCACT CTATCTCC /3InvdT/
Reactivator for P3	GGAGATAG AGTGGATGATCAA GAG TTT /3InvdT/
Rec-WkP3-mut	TCGTTGATCATCCACT T CTCAC
Rec-PCRP3-mut	TCGTTGATCATCCAC

Recording primers for all other tests on origami

Name	Sequence
Rec-WkP1	ACCTACCTAGCAATCT T CTCAC
Rec-WkP2	CATCTCTCAGCTTTCT T CTCAC
Rec-WkP3	CTCTTGATCATCCACT T CTCAC
Ref-WkP1	CCATACTCGACATCTT T CTCAC
Ref-WkP2	ACTCCCTTCGATACTT T CTCAC
Hex-rec-WkPr1	ACCTACCTAGCAATCT T CTCAC
Hex-rec-WkPr2	CATCTCTCAGCTTTCT T CTCAC
Hex-rec-WkPr3	CTCTTGATCATCCACT T CTCAC
Hex-rec-WkPr4	CTTCATCCTTACACAT T CTCAC
Hex-rec-WkPr5	CAAACCCCTTCTTCAT T CTCAC
Hex-rec-WkPr6	CTCCACCACTATTACT T CTCAC
Hex-rec-WkPr7	CCCTCACAATAGACAT T CTCAC

PCR primers for amplification of records

Name	Sequence
Rec-PCRPr1	ACCTACCTAGCAATC
Rec-PCRPr2	CATCTCTCAGCTTTC
Rec-PCRPr3	CTCTTGATCATCCAC
Ref-PCRPr1	CCATACTCGACATCT
Ref-PCRPr2	ACTCCCTTCGATACT
Hex-rec-PCRPr1	TTAATT ACCTACCTAGCAATC
Hex-rec-PCRPr2	TTAATT CATCTCTCAGCTTTC
Hex-rec-PCRPr3	TTAATT CTCTTGATCATCCAC
Hex-rec-PCRPr4	TTAATT CTTCATCCTTACACA
Hex-rec-PCRPr5	TTAATT CAAACCCCTTCTTCA
Hex-rec-PCRPr6	TTAATT CTCCACCACTATTAC
Hex-rec-PCRPr7	TTAATT CCCTCACAATAGACA

Supplementary Table 2: **Other origami-based probes, primers, and strands**. APR probes for click-chemistry linkage, as well as recording and PCR primers and other strands are listed. These are used in conjunction with those of Supplementary Table 1.

Supplementary Note 1: Probe Design

Continuously creating new DNA molecules that indicate proximity posed several challenges. The most challenging single performance objective was to allow the copying of an arbitrary sequence (i.e., the Half-record) and its isothermal release, a thermodynamically difficult task since a long duplex is quite stable. (A typical 16 nt duplex in this context spontaneously dissociates on average only every 20 years!) The solution presented here is to form the template sequence in a relatively stable hairpin stem (Supplementary Figure 2a), such that the stem can be opened for template copying but would thermodynamically "prefer" to be closed. Utilizing the energy bound in the phosphate bonds of free dNTPs, a displacing polymerase is used to drive the forward reaction of opening the stem and copying the template to the stopper position. A strand displacement reaction then stochastically displaces the extension from the stem. A careful balance of bulges in the sequence biases the system toward a closed stem and free, single stranded primer extension.

To demonstrate this bias, a thermodynamic prediction (using NUPACK[1]) of all possible nucleotide-nucleotide interactions (excluding "knot"-forming configurations) in the current design was calculated. Based largely on Santa Lucia and Hicks data,[3] they are predictions of the relative time spent in different states, but not the rates at or pathways by which they change. These are thus relevant under the assumption that the timescale of APR reaction is much longer than the timescale of investigating these states.

The interaction of the simplest probe, with an "Sp9" stopper of length ~1 nt in the hairpin loop, and a Half-record, is shown in Supplementary Figure 1a. The hairpin stem is predicted to be predominantly open and the primer extension predominantly hybridized - the opposite of the ideal situation. A small but important improvement is made by adding a short, strong stem below the stopper, increasing the rate at which stem closure begins by more closely co-localizing the stem template and complement (Main Text Figure 1b). In Main Text Figure 1c, the probe geometry used in Main Text Figures 3a and c, 4c, 5, and 6 is shown, with sequences outlined in Supplementary Figure 2. An asymmetrical T nucleotide bulge in the primer is more de-stabilizing than the synthetic stopper across from a T nucleotide, and NUPACK predicts the (blue) stem predominantly shut and the (purple) primer extension single stranded, as desired. Further improvements in performance were generated by tightening the hybridization at the stopper, using a synthetic base pair (Iso-dC/dG, IDT) (Main Text Figure 1d). Here, there is a symmetry in the reflecting boundaries of strand displacement, and therefore a symmetry in the probability profile. Adding a T-bulge to this system (Main Text Figure 1e) (or a weaker modification: a phosphorothioate bind in the primer - see main text description and Main Text Figure. 2, though this is more difficult to simulate in NUPACK) renders our highest-performing probe, used in Main Text Figure 3b.

The second challenge was to create Full-record molecules based on Half-record proximity. For this, a short palindromic sequence was inserted at the end of the extended Half-record (Main Text Figure 2b). When two Half-records are available for binding, they can bind each other at the 3' palindromic regions and use each other as a template for extension. Many palindrome lengths and sequences were investigated (not shown). Four-nucleotide sequences resulted in slower cycling due to weak interaction, and 8 nt sequences sometimes allowed Half-records to form hairpins and extend on themselves. Because a minimum of 3 nt are required for a hairpin loop itself, a 6 nt palindrome leaves little opportunity for intra-molecular pairing and extension. A strong (high C/G), 6 nt palindromic sequence yielded the best overall performance.

The entire APR cycle therefore had three apparent potential kinetic bottlenecks: Hybridization of the primer 3' end was slowed by a combination of the primer T nucleotide bulge and the stem complement, displacement of the primer extension (Half-record) from the stem was slowed by the stopper bulge, and Half-record pairing and extension required the availability and transient hybridization of two palindrome sequences in the presence of a polymerase. Because Half-records reached a steady-state level quickly compared to Full-record production (Main Text Figure 3b), single-stranded Half-record availability and pairing appeared to be slower processes. (Increasing the primer T nucleotide bulge to TT did make Half-record production the overall bottleneck and slowed net Full-record production.) Increasing spacer lengths significantly also slowed Full-record production, in part because of a diluting effect on the reactive palindrome pair.

Supplementary Note 2: Origami protection from displacing polymerases

Because they can be programmed to organize a complex set of elements on nm length scales, DNA origami nanostructures represent ideal testbeds for evaluating APR. The strand-displacing polymerase (here, Bst) necessary for APR function can damage origami structures by extending and displacing the component strands, however. We have found that binding origami to a mica surface protects origami from damage and enables experiments in Main Text Figures 4, 5, and 6.

When origami were incubated with Bst in solution, and then deposited on mica (Supplementary Figure 7a), only free DNA strands were visible under Atomic Force Microscopy (AFM) and no complete origami were found. Origami have been used as a testbed for super-resolution imaging and other techniques in the lab., and the typical method of attaching origami to a glass surface utilized layers of biotinylated Bovine Serum Albumin (BSA) to passivate the surface, streptavidin to hold both the BSA and the origami, and origami with biotinylated strands incorporated (Supplementary Figure 7b). This forms a layer perhaps 10 nm thick between the rigid glass and the 2.5 nm-thick origami. When origami held in this manner are augmented with Cy3-labeled staple strands and imaged with a fluorescent microscope, they cannot withstand the effects of Bst, as evidenced by the loss of Cy3 strands (Supplementary Figure 7c). Origami is commonly held onto a mica surface for AFM imaging, wherein free Mg^{2+} cations attract the negatively-charged origami to surface in a stable and presumably very close association (Supplementary Figure 7d). Origami held in such a manner before a long period of Bst incubation are much more stable (Supplementary Figure 7e). Fluorescence imaging confirms the longer lifetime of Cy3-labeled staple strands on mica-stabilized origami (Supplementary Figure 7f). We hypothesized that the protection against polymerase results from the manner in which origami binds mica tightly, perhaps inhibiting the polymerase from accessing origami staple 3' ends or displacing downstream staples.

For the atomic force microscope (AFM) test of mica-bound origami described here, a 5 μl of origami solution at 1 nM in TAE/Mg was deposited to a small (~1 cm by 1 cm) mica piece, fleshly cleaved and prewet with 20 μl of TAE/Mg. Extra staples were washed away by adding 20 μl of fresh buffer, mixing and taking out 20 μl 6 times, and then the buffer was exchanged to 1x ThermoPol buffer by washing with the buffer similarly 6 times. After taking out additional 20 μl of buffer on top of the mica piece, a 20 μl solution containing 0.8 units/μl of Bst polymerase and 100 μM dNTP in 1x ThermoPol buffer was added and incubated for ~2.5 hr at room temperature. AFM images were then taken in fluid tapping mode with a Multimode AFM (Veeco Metrology Group) using a Nanoscope V controller. Silicon nitride cantilevers with 2 nm radius silicon tips were used (SNL probes from Bruker).

For the test-tube incubation test, origami was purified by agarose gel electrophoresis (1% agarose in TAE/Mg, 100 V for 2 hr in an ice bath) and gel extraction spin columns (Freeze and Squeeze from Bio-Rad, Cat. No. 732-6166), where gel pieces were crushed, frozen at -20°C for 5 min, then spun at 450 g for 10 min at room temperature. A 40 μl solution containing ~0.3 nM purified origami (the concentration of purified origami was approximated by measuring the absorbance at 260 nm with an extinction coefficient 113,743,227 /M/cm, treating origami roughly as double-stranded m13), 0.8 units/μl of Bst polymerase and 100 μM dNTP in 1x ThermoPol buffer was incubated at 37°C for ~1 hr before 20 μl of the solution was deposited on freshly cleaved mica for AFM imaging.

For the fluorescence measurement experiments, a flow chamber system was used in the same way as described for APR recording reactions, except in the step of incubating with Bst the primers were omitted from the solution. For the glass surface test, a piece of glass coverslip (No. 1.5, from VWR, Cat. No. 48393 251) was used in the place of mica when constructing a flow chamber and treated as follows: after washing the chamber 3 times with 60 μl buffer A (10 mM Tris-HCl, 100 mM NaCl, 0.05% Tween 20, pH 7.5), 30 μl of biotin-labeled BSA (Sigma-Aldrich, Cat. No. A8549) solution (1 mg/ml in buffer A) was added and incubated for 2 min, followed by washing 3 times with 60 μl of buffer A, incubating with 30 μl of streptavidin (Invitrogen, Cat. No. S-888) solution (0.5 mg/ml in buffer A) for 2 min and washing 2 times with 60 μl buffer A and 2 times with 60 μl buffer B (5 mM Tris-HCl, 10 mM $MgCl_2$, 1 mM EDTA, 0.05% Tween 20, pH 8), before the origami solution was added. The fluorescence images were taken by Leica DMI6000B in TIRF mode with a 561 nm laser and a 100x oil immersion lens (numerical aperture 1.47). For the fluorescence measurements with mica, the mica piece needed to be cleaved very thin (~0.1-0.2 mm) to allow optical transmission similar to glass. Fluorescence images were analyzed with ImageJ.

Supplementary Note 3: The Worm-Like Chain model applied to probe reach

The Worm-Like Chain (WLC) model is often used to describe the physical behavior of polymers, treating polymers as continuously flexible chains.[4] The model has often been applied to nucleic acid molecules, e.g., for modeling elastic properties[5,6] and estimating persistence length.[7,8] Here, we use the WLC model to estimate the expected reach distance of a probe and attached Half-record.

We consider the end-to-end distances of the double-stranded and single-stranded portions of a probe (Main Text Figure 4b). For simplicity, we ignore the thickness of a DNA helix and the short linker at the bottom of a probe. The synthetic stopper and opposite T nucleotide are a mismatch pair but treated as part of the double-stranded part and counted as one pair. The palindrome (6 nucleotides) at the end of the single-stranded portion, where a Half-record binds a neighboring Half-record, is counted as 3 single-stranded nucleotides per side. The maximum reach distance per probe is calculated as the sum of the distances from the double-stranded part and from the single-stranded part. Since the linker under the double-stranded part and the phosphate backbone connecting the double-stranded and single-stranded parts would give orientational flexibility, we assume that the whole range of distances shorter than the max reach distance can be covered.

With a persistence length (~50 nm)[9] longer than the contour length (~6-12 nm at 0.34 nm per base pair), the double-stranded part of a probe can be treated as a rod with a narrow range of end-to-end distance. The root-mean-square (RMS) end-to-end distance R of the double-stranded part can be estimated by equation 1, based on the WLC model:[4]

$$\sqrt{\langle R^2 \rangle} = \sqrt{2L_{\mathrm{p}}L\left[1 - \frac{L_{\mathrm{p}}}{L}(1 - e^{-L/L_{\mathrm{p}}})\right]}, \tag{1}$$

where Lp is the persistence length and L is the contour length of a polymer. The calculated RMS end-to-end distances of the dsDNA portion with the spacer length 0, 12 and 18 nt are 6.0, 9.9, and 11.8 nm, respectively.

The single-stranded portion, with a persistence length (~0.75-2 nm)[10] much shorter than the contour length (~5-16 nm; 0.58 nm per nucleotide[10]), is expected to exhibit a more flexible behavior, with a wide range of end-to-end distances. We estimate the probability density of the end-to-end distance based on equation 2:

$$P(r,t) = 4\pi C \frac{r^2}{(1-r^2)^{9/2}} \exp\left[-\frac{3t}{4}\frac{1}{(1-r^2)}\right], \tag{2}$$

where r is the end-to-end distance (R) normalized to the contour length L (i.e., r = R/L), t is the contour length (L) in multiples of the persistence length Lp (i.e., t = L/Lp), and C is a normalization constant.[11]

By adding the two distances of the double-stranded and single-stranded parts, we estimate the probability densities of the total reach distance of a probe (Main Text Figure 8). The end-to-end distance of the double-stranded part was treated as fixed at the RMS distance, i.e., was used to "shift" the probability curves of the single-stranded part to longer lengths. The probability densities of the single-stranded part exhibit Gaussian-like distributions, and here we only take the right-hand-side of the curve for the consideration of the reach distance (again, the shorter distance is covered by the orientational flexibility of a probe). Each curve was normalized to have a common maximum (relative probability 1 at peak) for comparison between different spacer lengths.

To predict the pairwise reach distances (and compare them with the experimental data shown in Main Text Figure 4c), one would have to consider not only the combinatorial probabilities of the distances of the two probes, but also the interaction kinetics between two probes depending on the distance and corresponding effective volume for interactions, which is beyond the scope of this simple calculation. Nonetheless, it is notable that the overall shapes of the curves for a single probe match qualitatively well with the experimental data for pairwise interactions and that the critical distances (where the transition between high probability and low probability occurs) are in close agreement with roughly the halves of the critical transition distances of the pairwise data (Main Text Figure 4c).

Supplementary Note 4: Mathematica code used in basic graph drawing

Wolfram Mathematica (v10.3.1.0) was used to plot graphs given a set of connectivities. The GraphPlot command seeks to place vertices such that they are "well laid-out." The default method for laying out these vertices is to apply the "Spring-ElectricalEmbedding" physical model, which models each vertex as an electrical charge (thereby repelling all other vertices) and each connection as a spring (thereby attracting other connections with a force proportional to stretched distance), and calculates the equilibrium positions of the vertices. For the ConnectionList example below, Graphplot draws a 7-vertex hexagon geometry with labeled vertices. This is the code used in the Main Text Figure 5 reconstructions.

```
ConnectionList = {1 -> 2, 1 -> 3, 1 -> 4, 1 -> 5, 1 -> 6, 1 -> 7, 2 -> 3, 3 -> 4, 4 -> 5, 5 -> 6, 6 -> 7, 7 -> 2}

GraphPlot[ConnectionList, VertexRenderingFunction -> ({White, EdgeForm[Black], Disk[#, 0.1], Black, Text[#2, #1]} &)]
```

Supplementary References

1. Zadeh, J. N. *et al.* Nupack: analysis and design of nucleic acid systems. *Journal of computational chemistry* **32**, 170–173 (2011).
2. Jungmann, R. *et al.* Multiplexed 3d cellular super-resolution imaging with dna-paint and exchange-paint. *Nature methods* **11**, 313–318 (2014).
3. John SantaLucia, J. & Hicks, D. The thermodynamics of dna structural motifs. *Annual Review of Biophysics and Biomolecular Structure* **33**, 415–440 (2004). PMID: 15139820.
4. Flory, P. J. *Statistical mechanics of chain molecules* (Interscience Publishers, New York, 1969).
5. Marko, J. F. & Siggia, E. D. Stretching DNA. *Macromolecules* **28**, 8759–8770 (1995).
6. Wang, M. D., Yin, H., Landick, R., Gelles, J. & Block, S. M. Stretching DNA with optical tweezers. *Biophysical journal* **72**, 1335 (1997).
7. Bouchiat, C. *et al.* Estimating the persistence length of a worm-like chain molecule from force-extension measurements. *Biophysical journal* **76**, 409–413 (1999).
8. Abels, J., Moreno-Herrero, F., Van der Heijden, T., Dekker, C. & Dekker, N. Single-molecule measurements of the persistence length of double-stranded rna. *Biophysical journal* **88**, 2737–2744 (2005).
9. Rivetti, C., Guthold, M. & Bustamante, C. Scanning force microscopy of DNA deposited onto mica: Equilibration versus kinetic trapping studied by statistical polymer chain analysis. *Journal of molecular biology* **264**, 919–932 (1996).
10. Smith, S. B., Yujia, C. & Bustamante, C. Overstretching b-dna: the elastic response of individual double-stranded and single-stranded dna groups. *Science* **271**, 795–799 (1996).
11. Thirumalai, D. & Ha, B.-Y. Statistical mechanics of semiflexible chains: A meanfield variational approach. *arXiv preprint cond-mat/9705200* (1997).
